# Predicting Approximate Clinically Effective Doses in Oncology Using Preclinical Efficacy and Body Surface Area Conversion: A Retrospective Analysis

**DOI:** 10.3389/fphar.2022.830972

**Published:** 2022-04-26

**Authors:** Robert J. Griffin, Ethan Avery, Cindy Q. Xia

**Affiliations:** Takeda Pharmaceuticals, Cambridge, MA, United States

**Keywords:** oncology, effective dose, body surface area, preclinical efficacy, tumor xenograft

## Abstract

The correlation between efficacious doses in human tumor-xenograft mouse models and the human clinical doses of approved oncology agents was assessed using published preclinical data and recommended clinical doses. For 90 approved small molecule anti-cancer drugs, body surface area (BSA) corrected mouse efficacious doses were strongly predictive of human clinical dose ranges with 85.6% of the predictions falling within three-fold (3×) of the recommended clinical doses and 63.3% within 2×. These results suggest that BSA conversion is a useful tool for estimating human doses of small molecule oncology agents from mouse xenograft models from the early discovery stage. However, the BSA based dose conversion poorly predicts for the intravenous antibody and antibody drug conjugate anti-cancer drugs. For antibody-based drugs, five out of 30 (16.7%) predicted doses were within 3× of the recommended clinical dose. The body weight-based dose projection was modestly predictive with 66.7% of drugs predicted within 3× of the recommended clinical dose. The correlation was slightly better in ADCs (77.7% in 3×). The application and limitations of such simple dose estimation methods in the early discovery stage and in the design of clinical trials are also discussed in this retrospective analysis.

## Introduction

Prediction of human clinical effective dose from preclinical data enables two very important clinical activities. First is the preparation of an adequate amount of drug material to support the initial clinical dose escalation. Preparation of an insufficient amount will seriously interrupt and delay phase 1 studies while preparation of a large excess is costly and usually of no long-term value as most oncology trials fail. Second is the selection of the dose escalation cohorts. Knowing the safe starting dose and even a rough predicted effective dose allows the most efficient dose escalation study design with potential for significant cost and time savings. In addition, knowing the likely clinical dose escalation allows for the early preparation of the appropriate pill or capsule strengths. Typically, dose prediction activities occur just prior to first-in-human clinical studies when the preclinical data set is robust. Therefore, exposure and allometry-based approaches to prediction of human pharmacokinetics and clinical doses are usually scientifically appropriate and achievable at the late discovery stage.

The predicted human dose also has great value in the determination of dose feasibility. Dose feasibility is the likelihood that the efficacious exposure can be delivered in a clinically acceptable formulation. For oral drugs delivered as a pill or capsule this essentially means achieving an acceptable pill burden. Acceptable pill burden will vary by indication and depends on the extent to which the product delivers an unmet need, how patient compliance is compromised, excipient content and how competitor dosages will impact market share. For oral drugs delivered as a solution (gel caps etc) or intravenous and subcutaneous drugs, solubility, and the ability to deliver enough drug in an acceptable volume also becomes important. Determination of dose feasibility is of greatest value early in the drug discovery stage in order to avoid further development of drugs that would have a high risk of problematic or unacceptable clinical doses. In the late drug discovery stage, human dose predictions can be of critical value in the selection among leads for progression to clinical evaluation. Unfortunately, exposure-based dose prediction methods require significant preclinical data sets that are usually not fully available until drug candidates reach a more advanced stage.

An alternative to exposure-based dose predictions are the methods using either body surface area (BSA) based dose conversion for small molecules or body weight-based dose conversion for antibodies. These methods simply require an efficacious dose in a relevant and predictive animal model and the conversion of that dose to the human equivalent. This approach is recommended by FDA for the calculation of safe human starting dose from nonclinical NOAEL doses, but importantly, not for estimation of pharmacologically active doses (PAD) (CDER, 2005, European Medicines Agency, 2013). Regan-Shaw made a case for expanding use of BSA methods to the prediction of human efficacious doses (Reagan-Shaw et al., 2007). Blanchard and Smoliga revisited the concept and rightly pointed out that the underlying principle that BSA predicts physiological parameters is merely correlative rather than predictive, and that the FDA’s method to use BSA to initiate a safe starting dose actually estimates a dose that is likely to be in the safe range with a 10 × safety factor added. They also point out that supporting literature on BSA based drug dose predictions is sparse and questionable (Blanchard and Smoliga 2015). Their point that BSA-based doses are too imprecise for direct use as human clinical doses due to safety concerns is also fully justified. Redlarski et al. (2016) also further discussed the use, limitations and alternative calculation methods for body surface area correlations.

However, a correlative prediction of human doses is still of value in drug discovery and development. Despite the simplicity of the method, the accuracy and utility has not been thoroughly assessed. Although it is easy to find retrospective examples where this method was effective, there are minimal data on the success to failure rate. In addition, there is an understandable reluctance to rely on simple dose-based methods for dose prediction without validation.

In this report, the ability to predict current clinical doses using published *in vivo* preclinical effective doses was retrospectively evaluated for a large set of marketed oncology agents.

## Materials and Methods

### Clinical Doses

Current recommended clinical doses were obtained from FDA approved package inserts. In recognition that recommended doses change over time or vary by indication all clinical doses used in calculations are shown in the supplemental data tables. Where clinical dose recommendations are provided as a range, the average dose was used.

### Nonclinical Effective Doses

Effective doses in nonclinical studies were obtained from the literature. Searches were conducted using combinations of the drug name(s), mouse, human, model, xenograft, and antitumor. Mouse efficacy results from human-derived tumor xenograft models were used in all cases. Syngeneic mouse models are typically used for immune-oncology indications but were excluded from the dataset due to potential human *vs.* mouse tumor disconnects. Given the potential variability from what defines an efficacious response as well as variations in study designs, the following rules were followed for the literature assessment to minimize any investigator bias.

1) The most efficacious mouse doses in a sensitive model were used except where a large increase in dose resulted in a small increase in response, the lower dose was chosen
2) Efficacious dose was as defined by the reference authors as significant and for the most part was TGI (% tumor growth inhibition), T/C (treated tumor volume over control tumor volume), or mean survival. Stasis or tumor regression in xenograft models were preferred but tumor growth inhibition of ≥60% was also considered a lower end of efficacious response (Wong et al., 2012).
3) The preclinical tumor model and route of administration that was most similar to the clinical indication was used if possible. This was often not possible as clinical indications were defined in the clinic after preclinical proof of concept and subcutaneous (SC) or intraperitoneal (IP) dosing is often used as a more convenient preclinical model for intravenous (IV) drugs. In situations where nonclinical and clinical data on the same tumor was not available, inappropriate predictions were avoided such as systemic to CNS predictions (blood brain barrier may be confounding) or hematological to solid tumor predictions (tumor penetration may be confounding). Clinical and nonclinical tumor and clinical and nonclinical routes of administration are summarized in the supplemental tables for all data points.
4) Nonclinical single agent or combination dosing was matched with the equivalent clinical regimen where possible but many clinical oncology agents are dosed in combination while published nonclinical preclinical work is usually performed as single agent.
5) Nonclinical efficacy data that was clearly obtained only at an MTD was not used to avoid the potential for saturated response (no dose response).
6) All doses were calculated as the total dose administered on Day 1 of dosing over a 24-h period. Therefore, differences in intermittent dosing schedules were not factored into the calculations.
7) The first study found in the literature that met these requirements was selected before the dose was compared to the clinical dose with no further searching for additional results (in most cases there was only one appropriate study). This approach was selected to minimize potential for a bias toward studies that support the hypothesis with the understanding that it could result in some inappropriate comparisons in the data set and a reduced overall predictivity.
8) Some oncology drugs have multiple indications. Only one indication per drug was included in the analysis with preference given to the indication with the most similar tumor type and similar route of administration in the non-clinical studies.

### BSA Based Analysis

Mouse efficacious doses in mg/kg were converted to mg/m^2^ and then converted to the equivalent human effective dose in mg/kg using the standard conversion factors (Nair and Jacob 2016). In examples where published doses were not body weight normalized, 70 kg was used for human body weight and 25 g was used for mouse body weight.

$$\text{Mouse dose in mg/kg×}3={\text{mouse dose in mg/m}}^{2}$$

$${\text{Mouse dose in mg/m}}^{2}\text{÷}37=\text{human dose in mg/kg}$$

Data sets were divided into intravenous large molecule drugs (antibodies and antibody-drug conjugates), and small molecule drugs (IV or oral [PO]). Human effective doses were plotted versus predicted human dose in Excel on a log-log scale. The percentage of individual predictions that were with 3-fold (3×) and 2-fold (2×) of recommended clinical doses were also calculated.

### Body Weight-Based Analysis

For large molecules prediction from a direct conversion of nonclinical dose to clinical dose was assessed. For example 2 mg/kg in mouse = 2 mg/kg in human.

## Results

The approved drugs used in the correlation analyses, the nonclinical and clinical doses of these drugs, routes of administration, and the corresponding references are presented in the supplemental data tables along with a summary of the tumor models and indications. SupplementaryTable S1 (Intravenous Small Molecule Oncology Drugs), Supplementary Table S2 (Oral Small Molecule Oncology Drugs), Supplementary Table S3 (Antibody and Antibody Drug Conjugate Oncology Drugs), and Supplementary Table S4 (Small molecule immune and endocrine modulators).

**FIGURE 1:**
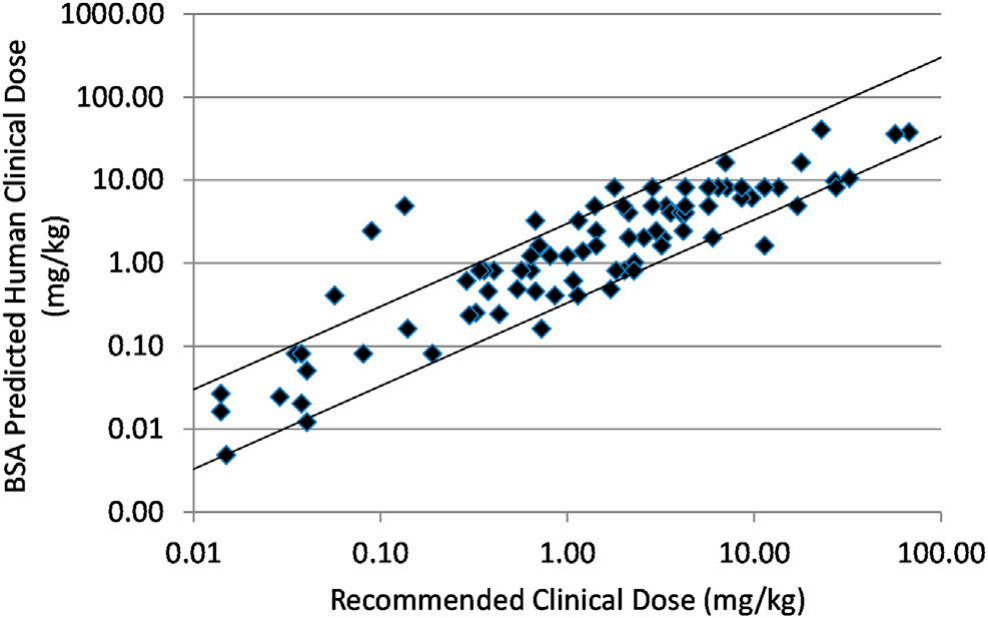
Relationship between recommended clinical doses and clinical doses predicted from BSA scaling of mouse effective doses for ninety marketed small molecule, intravenous or oral oncology drugs. Solid lines indicate plus and minus 3-fold from precise prediction.

In Figure 1 prediction of human effective dose by BSA conversion for a set of 90 small molecule intravenous or oral oncology drugs is shown. The relationship was predictive with 85.6 and 63.3% of the compounds falling within three- and two-fold of the actual recommended clinical dose, respectively.

**FIGURE 2:**
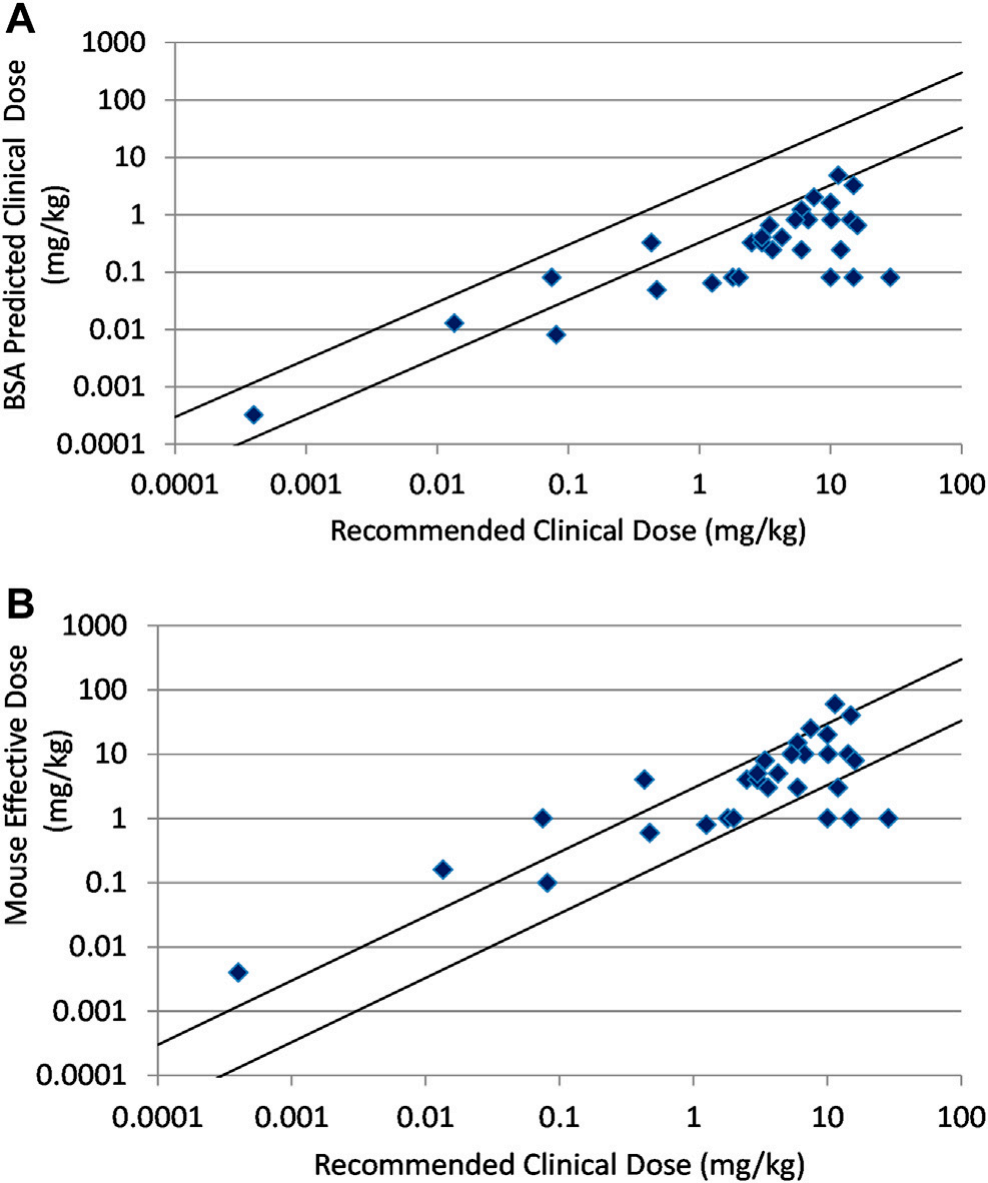
**(A)** Relationship between recommended clinical doses and clinical doses predicted from BSA scaling of mouse effective doses for thirty marketed, large molecule oncology drugs. **(B)** Relationship between recommended clinical doses and mouse effective doses for thirty marketed, large molecule oncology drugs. Solid lines indicate plus and minus 3-fold from precise prediction.

In Figure 2A the correlation between human clinical dose and the clinical dose predicted by BSA conversion of mouse effective doses is shown for a set of thirty large molecule oncology drugs. The relationship was poorly predictive with predictions for 25 of 30 compounds falling beyond three-fold of actual. For many of these the recommended clinical dose was much greater than three-fold the BSA predicted dose. The BW predicted or direct correlation between human clinical dose and the mouse effective doses in mg/kg is shown for the same set of thirty large molecule drugs in Figure 2B. This simpler analysis provides a better correlation with only 10 of 30 compounds showing a greater than 3× difference between the human dose and mouse dose in mg/kg.

In Table 1 the numerical results of these analyses are summarized with additional subdivisions of the test sets. The small molecule drugs were subdivided into 35 intravenous and 55 oral drugs. The BSA based approach was predictive of clinical doses for both sets although the results were marginally better for oral than intravenous drugs. For IV drugs BSA predicted within 3× and 2× of predicted 82.8 and 57.1% of the time and for oral drugs 87.2 and 67.3%. Besides the above mentioned 90 anticancer small molecules, there were nine immune targeted or hormone targeted anticancer small molecules that were analyzed separately due to the potential confounding interactions with the mouse immune and endocrine systems. For these 9 compounds prediction was poor (only 22.2 and 33.3% predicted within 2× and 3×, respectively). These agents were removed from the main small molecule data and the data are shown in Supplementary Table S4.

**TABLE 1:** Prediction of clinical doses by BSA and BW based approaches for oncology drugs.

Class	Sub-classification	Total compounds	% Within 2× by BSA	% Within 3× by BSA	% Within 2× by BW	% Within 3× by BW
Small Molecule	IV	35	57.1	82.8	NA	NA
Small Molecule	PO	55	67.3	87.2	NA	NA
Small Molecule	IV + PO	90	63.3	85.6	NA	NA
Small Molecule	IO/HT	9	22.2	33.3	NA	NA
Large molecule	mAB + ADC	30	13.3	16.7	56.7	66.7
Large molecule	ADC	9	22.2	22.2	77.7	77.7
Large molecule	mAb	21	9.52	14.2	47.6	61.9
Large molecule	Checkpoint Inhibitor	3	0	0	66.7	100

ADC, antibody-drug conjugate; BSA, body surface area; BW , body weight; HT, hormonal therapy; IV, intravenous(ly); IO, immuno-oncology; mAb = monoclonal antibody; NA, not applicable; PO, per-oral(ly).

In the large molecule data set, there were three checkpoint inhibitors. All three checkpoint inhibitors were more than 3× outside of the actual dose by BSA prediction, but direct dose prediction using body weight was within 3× (100%). This observation may be the result of the very small number of replicates for checkpoint inhibitors. The large molecule data set could also be divided into antibody and ADC drugs. ADC drugs showed superior prediction using mg/kg comparison over antibodies with 77.7% of ADCs (6 out of 9 compounds) predicted within 2× and 3× while antibodies were predicted within 2 × 47.6% of the time and within 3 × 61.9% of the time. Apart from the three immune targeted checkpoint inhibitors which predicted well, there were three hormone targeted drugs and several more drugs that have immune components to their mechanism among the relatively small set of large molecules. These agents may require interaction with the mouse immune and endocrine systems that add a layer of complexity absent in the other more direct tumor targeting agents. It is therefore not surprising that the large molecule agents performed more poorly overall than the small molecules.

## Discussion

One of the major goals of the pharmaceutical DMPK scientist in the drug discovery and development arena is to predict the exposure-based behavior of drug candidates in humans using nonclinical models. This includes the prediction of pharmacokinetics (PK), drug-drug interactions and the doses that are needed for clinical response. There are well established but extensive data and knowledge-based methods for such predictions that include the physiological-based (PB)-PK-pharmacodynamic (PD) modeling approach. However, simpler dose-based methods are useful during the earlier discovery phase for dose feasibility assessment. Best known empirical methods to estimate the human doses from preclinical doses are using BSA or BW as a correction factor. This simple mathematical calculation is often used to quickly scale doses between species and is used more formally as part of one of the FDA recommended processes to determine human safe starting dose (CDER 2005, European Medicines Agency, 2013). However, no formal assessment of the reliability of these methods is available for pharmacological dose. In this report the reliability was retrospectively assessed for oncology drugs.

Suitable preclinical data was found for a total of 90 marketed small molecule oncology drugs. The body surface area conversion method resulted in a strong prediction as shown in Figure 1 and Table 1 with 85.6% of the drugs predicted within 3× and 63.3% within 2×. Dividing the data set into intravenous and oral route of administrations, reveals that the prediction is slightly weaker but probably not meaningfully weaker for IV drugs. A small set of immune targeted and hormone targeted agents (*n* = 9) were excluded from the main data set. These mechanisms are more complex with effects on the mouse immune and endocrine systems that might be expected to reduce prediction. And in fact, these agents were poorly predicted with combined prediction rates of 33.3% within 3× and 22.2% within 2× (Supplementary Table S2). This observation supports the hypothesis that the best correlation in biology increases the probability of accurate prediction.

As seen in Figure 2A and Table 1, prediction using simple BSA conversion is clearly contraindicated for large molecule oncology drugs with only 16.7% of the predictions falling within 3× of the actual recommended clinical dose. It is also clear that preclinical efficacy predicts the clinical dose much better using a direct mg/kg comparison for these large molecule drugs (Figure 2B and Table 1). This is consistent with the recommendation in the FDA guidance document to use mg/kg conversion with intravascular administration of high molecular weight proteins (MW > 100 KDa). This poor prediction may result from human tumor xenografts that are not as optimized for large molecules. These models have been used and optimized for small molecules over decades with a demonstrated predictivity for small molecule oncology drugs, but this has not been as clearly demonstrated for large molecules (Wong et al., 2012). Or it may be indicative of an underlying difference in pharmacokinetic behavior between small and large molecule drugs. It is well known that unlike small molecules, the distribution and clearance of antibodies and ADCs and their resulting exposures is largely dependent on their interaction with the target receptors (target mediated drug disposition or TMDD) and the FcRn receptor that regulates endogenous antibody clearance (Prueksaritanont and Tang, 2012; Wang et al., 2008). Most antibodies in this evaluation are either humanized or human antibodies and may have TMDD occurring in patients but not in mice resulting from the human-specific binding. However, non-linear PK resulting from saturation of TMDD cannot fully explain the failure to predict clinical efficacious doses. Among 9 antibody and ADC drugs which had predicted doses more than 3× outside of the clinical dose range, bevacizumab and necitumumab have linear PK and the efficacious dose of margetuximab-CMBK was in the linear PK range although TMDD was observed at the low dose. Elotuzumab and ofatumumab have TMDD in patients and underestimated prediction from mice, alemtuzumab, inotuzumab ozogamicin, and loncastuximab tesirene-LPYL have TMDD in patients with high overprediction from mouse models (Ryman and Meibolm, 2017). In contrast, cetuximab and trastuzumab have TMDD but have relatively accurate dose predictions. Other factors related to TMDD that might impact the predictions include variations in the expression of the target receptors with disease progression or treatment and the affinity of the drug for the target receptor as well as the affinity for endogenous FcRn and potential competitive binding of M-Protein with FcRn from multiple myeloma patients such as elotuzumab and daratumumab (FDA approved package insert). In any event, even with 66.7% of the antibody based drugs predicted within 3× and 56.7% within 2× by body weight method, the relationship is modest and of limited usefulness for antibody-based drugs. There may still be an underlying and useful relationship relating nonclinical and clinical dose for antibody-based drugs, but a better method for making the dose predictions remains to be elucidated.

Among subsets of the large molecule data set, the ADCs predicted better than antibodies when using the BW-based approach with 77.7% prediction within 3× based on an *n* = 9. This intermediate result is possibly consistent with the hybrid nature of ADCs with their antibody delivery mechanism resulting in the release of a small molecule warhead. Checkpoint inhibitors were well predicted, but this is counter-intuitive given the additional complexity of human to mouse immune interactions in the xenograft models and may just be the result of the very low number of examples (*n* = 3). Interestingly, although the bispecific T-cell engager, blinatumomab, is an antibody, it has a molecular weight of 54.1 KDa which is below the 100 KDa cut-off proposed in the FDA guidance for using the body weight method. This bispecific showed good prediction using the BSA-based approach. This is only a single example but it will be interesting to see if the relationship holds as more lower molecular weight antibody fragment based drugs reach the market.

BSA conversion is clearly a useful method for predicting effective human small molecule dose ranges in oncology a significant percentage of the time, but what are the limitations? Prediction of human doses from preclinical doses using BSA normalization assumes that physiological, pharmacokinetic/pharmacodynamic and efficacy relationships scale roughly linearly with body surface area across species. In fact, there is considerable debate on the degree of correlation (Raegan-Shaw et al., 2007; Blanchard and Smoliga, 2015). Species differences in protein binding can result in differences in clearances but the effect on therapeutic dose should be counterbalanced by the change in free fraction at the active site (Smith et al., 2010). Large differences in metabolism, elimination or absorption across species may be rare but do occur and should impact the predictability of body surface area conversion. It can be difficult to find complete nonclinical and clinical data sets in the literature but closer examination of the stronger outliers in the small molecule data set provides some insight. The BSA predicted dose for axitinib was 36× higher than the clinical dose but the human oral bioavailability is 3.6× higher than in mouse (Committee for Medicinal Products for Human Use, 2012; Chen et al., 2013). This reported difference in bioavailability is insufficient to fully explain the discrepancy but is certainly contributing, especially if the bioavailability drops at the high dose used in the mouse model. The second most prominent outlier in the data set is Leustatin with a 27× over prediction of the human dose. Closer examination revealed that the tumor model used was later determined to be unrelated to hairy cell leukemia and that tumor models are generally not predictive for this indication (Den Otter et al., 2002; Drexler et al., 2003). These examples highlight the need for the greatest understanding of the properties of the compound, the animal model and the indication to maximize the correct inputs and appropriateness of the method. Because this methodology assumes rough similarity in all properties across species, which will not always be the case, it must be considered a statistical probability assessment. Therefore, other more complex and mechanistic methods such as mechanistic-based PK/PD/Efficacy or PBPK/PD modeling approaches should supersede BSA-based approaches when possible. Another potential limitation in this data set to consider is the absence of failed drugs due to the limited published non-clinical data. Drugs that did not reach the market are not represented in the data set and may not behave in this manner.

An obvious question to explore would be: does this methodology also extend to other therapeutic areas? Broad predictability of human tumor xenograft systems was confirmed for small molecule targeted and cytotoxic agents (Wong et al., 2012). Oncology has the benefit of using humanized models with a human tumor in the mice, and therefore having very similar target pharmacology in both the mouse and patient. This removes an important potential source of pharmacological variability over and above pharmacokinetic variability. Theoretically this method is therefore most likely to be of benefit in indications where the nonclinical target pharmacology is humanized or where the target pharmacology resides in an infective agent common to the preclinical models and humans (anti-bacterials, antivirals, antifungals, and anti-parasitics).

BSA based dose predictions should never be solely used to set human doses. The method provides a statistical probability of a range of PAD and it is too imprecise to support human safe dose estimation. BSA based approaches in a validated data set like small molecule oncology drugs can be useful in predicting likely clinical dose ranges to assess relative risk benefit and chance of success for multiple preclinical candidates. They can also supplement or replace problematic allometric or model-based predictions for the estimation of clinical effective dose ranges that combined with safe starting doses can then be used to design the most efficient clinical dose escalation protocols to test and define the actual clinical dose. Basically, BSA based methods are a tool in the toolbox that can be used for decision making and compound progression as long as the limitations are clearly understood and considered.

In conclusion, dose-based methodologies for predicting human clinical doses from preclinical data were assessed for oncology drugs. BSA-based approaches were predictive for small molecule oncology drugs, in particular for kinase inhibitors and cytotoxic agents, but prediction was poor for drugs with immune and endocrine components to their mechanisms. BSA conversion of doses was clearly inappropriate for large molecules. Direct mg/kg-based prediction was more relevant to large molecules with MW > 100 kDa and in particular ADCs. This approach is theoretically applicable to other therapeutic areas and if validated in other therapeutic areas may provide an easy estimate of clinical doses early in the drug discovery and development process to facilitate compound selection and risk management. Later in the drug development process, dose-based methods should be superseded by exposure- and mechanism-based methodologies whenever possible.

## Data Availability

The original contributions presented in the study are included in the article/Supplementary Material, further inquiries can be directed to the corresponding author.
