# Supplementary material for: Predicting Approximate Clinically Effective Doses in Oncology Using Preclinical Efficacy and Body Surface Area Conversion: A Retrospective Analysis
Source: Front Pharmacol. 2022 Apr 26;13:830972. doi: 10.3389/fphar.2022.830972 (PMC9087189; doi:10.3389/fphar.2022.830972)
Supplement: Supplementary file 1 [file DataSheet1.docx]

| Supplemental Table 1 Intravenous Antibody and Antibody Drug Conjugate Oncology Drugs | | | | | | | | |
| --- | --- | --- | --- | --- | --- | --- | --- | --- |
| **Agent** | **Clinical Dose/  Mechanism/  Indication** | **Clin Dose (mg/kg)** | **Mouse Efficacious Dose (mg/kg)** | **Predicted human Dose by BSA (mg/kg)** | **Xenograft Model/ Cell Line/ Preclinical Dose** | **Preclinical Data Source** | **BSA Ratio** | **mg/kg Ratio** |
| Alemtuzumab (Campath) | 30 mg/ CD52 mAb/ B-CLL | 0.43 | 4 | 0.32 | AML  (EVI1^High^ cells/ 4 mg/kg IV | Saito et al 2011 | 0.75 | 0.11 |
| ado-trastuzumab (Kadcyla) | 3.6 mg/kg/ HER2 ADC/ Breast | 3.60 | 3 | 0.24 | Breast  (MDA-MB-175-VII and KPL-4 cells)/ 3 mg/kg IV | Phillips et al 2013 | 0.07 | 1.20 |
| belantamab mafodotin-blmf (Blenrep) | 2.5 mg/kg/ BCMA mitotic inh ADC/ MM | 2.5 | 4 | 0.32 | MM  (H929 and OPM2 cells)/ 4 mg/kg IP | Tai et al 2014 | 0.13 | 0.63 |
| Bevacizumab (Avastin) | 5-10 mg/kg/ VEGF mAb/ CRC | 7.50 | 25 | 2.03 | CRC  (PDX)/ 25 mg/kg IV | Chiron et al 2014 | 0.27 | 0.30 |
| Blinatumomab (Blincyto) | 28 ug/ Bispecific CD19/CD3/ ALL | 0.0004 | 0.004 | 0.0003 | Lymphoma  (NALM-6 cells)/ 0.1 µg/25g IV | Dreier et al 2003 | 0.81 | 0.10 |
| Brentuximab vedotin (Adcetris) | 1.8 mg/kg/ CD30 ADC/ ALCL | 1.80 | 1 | 0.08 | ALCL  (Karpas 299 and L540cy cells)/ 1 mg/kg IV | Francisco et al, 2003 | 0.05 | 1.80 |
| Cetuximab (Erbitux) | 250 mg/m^2^/ EGFR mAb/  HN CRC | 6.76 | 10 | 0.81 | CRC  (GEO human carcinoma cells)/ 0.25 mg /25 g IV | Luo et al 2005. | 0.12 | 0.68 |
| Daratumumab (Darzalex) | 16 mg/kg/ CD38 mAb/ MM | 16.0 | 8 | 0.65 | MM  (UM9, L363-CD38, cl2.2, LME-1, and RPMI8226 cells)  8 mg/kg IV | Nijhof et al 2015 | 0.04 | 2.00 |
| Dinutuximab (Unituxin) | 17.5 mg/m2/ GD2 mAb/ Neuroblastoma | 0.47 | 0.6 | 0.05 | Neuroblastoma  (PDX)/ 15µg/25g IV | Barry et al 2019 | 0.10 | 0.79 |
| Elotuzumab (Empliciti) | 10 mg/kg/ SLAMF7 mAb/ MM (IO) | 10 | 1 | 0.08 | MM  (MM.1S GFP+Luc+ cells)/ 1 mg/kg IP | Kurdi et al 2018 | 0.01 | 10.00 |
| enfortumab vedotin-ejfv (Padcev) | 1.25 mg/kg/ Nectin-4 mAb Microtubule inh ADC/ urothelial | 1.25 | 0.8 | 0.06 | Bladder  (AG-B1 cells)/ 0.8 mg/kg IV | Challita-Eid et al 2016 | 0.05 | 1.56 |
| fam-trastuzumab deruxtecan-nxki (Enhertu) | 5.4 mg/kg/ HER2 mAb Topoisomerase inh ADC/ Breast | 5.40 | 10 | 0.81 | Breast  (BT-474 cells)/ 10 mg/kg IV | Conilh et al 2021 | 0.15 | 0.54 |
| Gemtuzumab ozogamicin (Mylotarg) | 3 mg/m^2^/ CD33 ADC/ AML | 0.08 | 0.1 | 0.01 | AML  (MV4-11 cells/ 0.1 mg/kg IV | Carr et al 2020 | 0.10 | 0.81 |
| Inotuzumab ozogamicin (Besponsa) | 0.5 mg/m2/ CD22 ADC/ ALL | 0.01 | 0.16 | 0.01 | BCL  (Ramos)/ 160 µg/kg IP | DiJoseph et al 2004 | 0.96 | 0.08 |
| Ipilimumab (Yervoy) | 3 mg/kg/ CTLA4 mAb/ Melanoma | 3 | 5 | 0.41 | Melanoma  (MC1)/ 5 mg/kg IV | Rosato et al 2018 | 0.14 | 0.60 |
| Loncastuximab Tesirene-LPYL (Zynlonta) | 0.075 mg/kg/ CD19 mAb Alkylating ADC/ BCL | 0.075 | 1 | 0.08 | BCL  (PDX)/ 1mg/kg IV | Zammarchi et al 2018 | 1.08 | 0.08 |
| Margetuximab-CMKB (Margenza) | 15 mg/kg/ HER2 Inh/ Breast | 15 | 1 | 0.08 | Breast (JIMT-1 cells)/ 1mg/kg IV | Nordstrom et al 2011 | 0.01 | 15.00 |
| Naxitamab-gqgk (Danyelza) | 3 mg/kg/ GD-2 mAb/ Neuroblastoma | 3 | 4 | 0.32 | Neuroblastoma  (LAN-1 cells)/ 100 µg/25g IV | Cheung et al 2012 | 0.11 | 0.75 |
| Necitumumab (Portrazza) | 800 mg/ EGFR mAb/ NSCLC | 11.4 | 60 | 4.86 | NSCLC  (A549 and NCI-1650 cells)/ 60 mg/kg IP | Samakoglu et al 2012 | 0.43 | 0.19 |
| Nivolumab (Opdivo) | 240 mg/ PD-1 mAb/  CRC/Gastric | 3.43 | 8 | 0.65 | Colon and gastric  (HT29 cells)/ 200 µg/25 g IV | Sanmamed et al 2015 | 0.19 | 0.43 |
| Obinutuzumab (Gazyva) | 1000 mg/ CD20 mAb/ CLL | 14.29 | 10 | 0.81 | MCL  (Z138 cells/ 10 mg/kg IV | Herting et al 2014 | 0.06 | 1.43 |
| Ofatumumab (Arzerra) | 2000 mg/ CD20 mab/ CLL | 28.57 | 1 | 0.08 | Lymphoma  (PDX)/  1 mg/kg IV | Barth et al 2012 | 0.00 | 28.57 |
| Olaratumab (Lartruvo) | 15 mg/kg/ PDGFR mAb/ Soft tissue sarcoma | 15 | 40 | 3.24 | Sarcoma  (A-204 cells)/ 40 mg/kg IP | Lowery et al 2018 | 0.22 | 0.38 |
| Panitumumab (Vectibix) | 6 mg/kg/ EGFR mAb/ CRC | 6.00 | 3 | 0.24 | CRC  (LIM1215)/  3 mg/kg IP | Taniguchi et al 2018 | 0.04 | 2.00 |
| Pembrolizumab (Keytruda) | 200-400 mg/ PD-1 mAb/ Multiple | 4.28 | 5 | 0.41 | Bladder/NSCLC  (PDX)/ 5mg/kg IP | Wang et al 2018 | 0.09 | 0.86 |
| Pertuzumab (Perjeta) | 420 mg/ HER2 mAb/ Breast | 6 | 15 | 1.22 | Breast (Calu-3 and KPL-4 cells)/15 mg/kg IP | Scheuer et al 2009 | 0.20 | 0.40 |
| Rituximab (Rituxan) | 375 mg/m^2^/ CD20 mAb/ NHL | 10.14 | 10 | 0.81 | Lymphoma (PH061/L cells/PDX)/10 mg/kg IP | Butler et al 2017 | 0.08 | 1.01 |
| sacituzumab govitecan-hziy (Trodelvy) | 10 mg/kg/ TRP2 ADC/ Breast and Urothelial | 10 | 20 | 1.62 | Endometrial  (Trop-2+ END(K)265 cells)/ 0.5mg/25g IV | Perrone et al 2020 | 0.16 | 0.50 |
| tafasitamab-cxix (Monjuvi) | 12 mg/kg/ CD19 mAb/ BCL | 12 | 3 | 0.24 | Lymphoma  (Raji and Ramos cells)/ 3mg/kg IP | Horton et al 2008 | 0.02 | 4.00 |
| Trastuzumab (Herceptin) | 2 mg/kg/ HER2 mAb/ Breast | 2.00 | 1 | 0.08 | Breast  (LCC6^HER2^ and MCF-7^HER2^ cells) 1 mg/kg IV | Warbuton et al 2004 | 0.04 | 2.00 |
| ADC: antibody-drug conjugate; ALCL: anaplastic large cell lymphoma; ALL: acute lymphocytic leukemia; AML: acute myeloid leukemia; BCL: B-cell lymphoma; BCMA: B cell maturation antigen; CLL: chronic lymphocytic leukemia; CRC: colorectal cancer; CTLA-4: cytotoxic T-lymphocyte–associated antigen 4; EGFR: epidermal growth factor receptor; HER2: human epidermal growth factor receptor 2; HN: head and neck; inh: inhibitor; IO: immune-oncology; IP: intraperitoneal(ly); IV: intravenous(ly); mAb: monoclonal antibody; MM: multiple myeloma; NHL: non-Hodgkin’s lymphoma; NSCLC: non-small cell lung cancer; PD-1: programmed cell death protein 1; PDGFR: platelet-derived growth factor receptor; PDX: patient-derived; VEGF: vascular endothelial growth factor receptor.  Barry WE, Jackson JR, Asuelime GE, Wu H-W, Sun J, Wan Z, et al. (2019). Activated natural killer cells in combination with anti-GD2 antibody dinutuximab improve survival of mice after surgical resection of primary neuroblastoma. Clin Cancer Res. 25(1):325-33. doi: 10.1158/1078-0432.CCR-18-1317.  Barth MJ, Hernandez-Ilizaliturri FJ, Mavis C, Tsai P-C, Gibbs JF, Deeb G, et al. (2012). Ofatumumab demonstrates activity against rituximab-sensitive and -resistant cell lines, lymphoma xenografts and primary tumour cells from patients with B-cell lymphoma. Br J Haematol. 156(4):490-8. doi: 10.1111/j.1365.2141.2011.08966.x.  Butler KA, Hou X, Becker MA, Zanfagnin V, Enderica-Gonzalez S, Visscher D, et al. (2017). Prevention of human lymphoproliferative tumor formation in ovarian cancer patient-derived xenografts. Neoplasia. 19(8):628-36. doi: 10.1016/j.neo.2017.04.007.  Carr MI, Zimmermann A, Chiu L-Y, Zenke FT, Blaukat A, Vassilev LT. (2020). DNA-PK inhibitor, M3814, as a new combination partner of Mylotarg in the treatment of acute myeloid leukemia. 10:127. doi: 10.3389/fonc.2020.00127.  Challita-Eid PM, Satpayev D, Yang P, An Z, Morrison K, Shostak Y, et al. (2016). Enfortumab vedotin antibody-drug conjugate targeting nectin-4 is a highly potent therapeutic agent in multiple preclinical cancer models. Cancer Res. 76(10):3003-13. doi: 10.1158/0008-5472.CAN-15-1313.  Cheung N-KV, Guo H, Hu J, Tassev DV, Cheung IY. (2012). Humanizing murine IgG3 anti-GD2 antibody m3F8 substantially improves antibody-dependent cell-mediated cytotoxicity while retaining targeting in vivo. Oncoimmunology. 1(4):477-86. doi: 10.4161/onci.19864.  Chiron M, Bagley RG, Pollard J, Mankoo PK, Henry C, Vincent L, et al. (2014). Differential antitumor activity of aflibercept and bevacizumab in patient-derived xenograft models of colorectal cancer. Mol Cancer Ther. 13(6):1636-44. doi: 10.1158/1535-7163.MCT-13-0753.  Conilh L, Fournet G, Fourmaux E, Murcia A, Matera E-L, Joseph B, et al. (2021). Exatecan antibody drug conjugates based on a hydrophilic ploysarcosine drug-linker platform. Pharmaceuticals. 14(3):247. doi: 10/3390/ph14030247.DiJoseph JF, Armellino DC, Boghaert ER, Khandke K, Dougher MM, Sridharan L, et al. (2004). Antibody-targeted chemotherapy with CMC-544: a CD22-targeted immunoconjuagte of calicheamicin for the treatment of B-lymphoid malignancies. Blood. 103(5):1807-14. doi: 10.1182/blood-2003-07-2466.  Dreier T, Baeuerle PA, Fichtner I, Grün M, Schlereth B, Lorenczewski G, et al. (2003). T cell costimulus-independent and very efficacious inhibition of tumor growth in mice bearing subcutaneous or leukemic human B cell lymphoma xenografts by a CD19-/CD3- bispecific single-chain antibody construct. J Immunol. 170(8):4397-402. doi: 10.4049/jimmunol.170.8.4397.  Francisco JA, Cerveny CG, Meyer DL, Mixan BJ, Klussman K, Chace DF, et al. (2003). cAC10-vcMMAE, an anti-CD30-monomethyl auristatin E conjugate with potent and selective antitumor activity. Blood. 102(4):1458-65. doi: 10.1182/blood-2003-01-0039.  Herting F, Friess T, Bader S, Muth G, Hölzlwimmer G, Rieder N, et al. (2014). Enhanced anti-tumor activity of the glycoengineered type II CD20 antibody obinutuzumab (GA101) in combination with chemotherapy in xenograft models of human lymphoma. Leuk Lymphoma. 55(9):2151-60. doi: 10.3109/10428194.2013.856008.  Horton HM, Bernett MJ, Pong E, Peipp M, Karki S, Chu SY, et al. (2008). Potent in vitro and in vivo activity of an Fc-engineered anti-CD19 monoclonal antibody against lymphoma and leukemia. Cancer Res. 68(19):8049-57. doi: 10.1158/0008-5472.CAN-08-2268.  Kurdi AT, Glavey SV, Bezman NA, Jhatakia A, Guerriero JL, Manier S, et al. (2018). Antibody-dependent cellular phagocytosis by macrophages is a novel mechanism of action of elotuzumab. Mol Cancer Ther. 17(7):1454-63. doi: 10.1158/1535-7163.MCT-17-0998.  Lowery CD, Blosser W, Dowless M, Knoche S, Stephens J, Li H, et al. (2018). Olaratumab exerts antitumor activity in preclinical models of pediatric bone and soft tissue tumors through inhibition of platelet-derived growth factor receptor α. Clin Cancer Res. 24(4):847-57. doi: 10.1158/1078-0432.CCR-17-1258.  Luo FR, Yang Z, Dong H, Camuso A, McGlinchley K, Fager K, et al. (2005). Correlation of pharmacokinetics with the antitumor activity of cetuximab in nude mice bearing the GEO human colon carcinoma xenograft. Cancer Chemother Pharmacol. 56(5):455-64. doi: 10.1007/s00280-005-1022-3.  Nijhof IS, Groen RWJ, Noort WA, van Kessel B, de Jong-Korlaar R, Bakker J, et al. (2015). Preclinical evidence for the therapeutic potential of CD38-targeted immuno-chemotherapy in multiple myeloma patients refactory to lenalidomide and bortezomib. Clin Cancer Res. 21(12):2802-10. doi: 10.1158/1078-0432.CCR-14-1813.  Nordstrom JL, Gorlatov S, Zhang W, Yang Y, Huang L, Burke S, et al. (2011). Anti-tumor activity and toxicokinetics analysis of MGAH22, an anti-HER2 monoclonal antibody with enhanced Fcγ receptor binding properties. Breast Cancer Res. 13(6):R123. doi: 10.1186/bcr3069.  Perrone E, Manara P, Lopez S, Bellone S, Bonazzoli E, Manzano A, et al. (2020). Sacituzumab govitecan, an antibody-drug conjugate targeting trophoblast cell-surface antigen 2, shows cytotoxic activity against poorly sifferentiated endometrial adenocarcinomas in vitro and in vivo. Mol Oncol. 14(3):645-56. doi: 10.1002/1878-0261.12627.  Phillips GDL, Fields CT, Li G, Dowbenko D, Schaefer G, Miller K, et al. (2013). Dual targeting of HER2-positive cancer with trastuzumab emtansine and pertuzumab: critical role for neuregulin blockade in antitumor response to combination therapy. Clin Cancer Res. 20(2):456-68. doi: 10.1158/1078-0432.CCR-13-0358.  Rosato RR, Dávila-González D, Choi DS, Qian W, Chen W, Kozielski AJ, et al. (2018). Evaluation of anti-PD-1-based therapy against triple-negative breast cancer patient-derived xenograft tumors engrafted in humanized mouse models. Breast Cancer Res. 20(1):108. doi: 10.1186/s13058-018-1037-4.  Samakoglu S, Deevi DS, Li H, Wang S, Murphy M, Bao C, et al. (2012). Preclinical rationale for combining an EGFR antibody with cisplatin/gemcitabine for the treatment of NSCLC. Cancer Genomics Proteomics. 9(2):77-92.  Sanmamed MF, Rodriguez I, Schalper KA, Oñate C, Azpilikueta A, Rodriguez-Ruiz ME, et al. (2015). Nivomumab and urelumab enhance antitumor activity of human T lymphocytes engrafted in Rag2-/-IL2Rγnull immunodeficient mice. Cancer Res. 75(17):3466-78. doi: 10.1158/0008-5472.CAN-14-3510.  Scheuer W, Friess T, Burtscher H, Bossenmaier B, Endl J, Hasmann M. (2009). Strongly enhanced antitumor activity of trastuzumab and pertuzumab combination treatment on HER2-positive human xenograft tumor models. Cancer Res. 69(24):9330-6. doi: 10.1158/0008-5472.CAN-08-4597.  Saito Y, Nakahata S, Yamakawa N, Kaneda K, Ichihara E, Suekane A, Morishita K. (2011). CD52 as a molecular target for immunotherapy to treat acute myeloid leukemia with high EVI1 expression. Leukemia. 25(6):921-31. doi: 10.1038/leu.2011.36.  Tai Y-T, Mayes PA, Acharya C, Zhong MY, Cea M, Cagnetta J, et al. (2014). Novel anti-B-cell maturation antigen antibody-drug conjugate (GSK2857916) selectively induces killing of multiple myeloma. Blood. 123(20):3128-38. doi: 10.1182/blood-2013-10-535088.  Taniguchi H, Baba Y, Sagiya Y, Gotou M, Nakamura K, Sawada H, et al. (2018). Biologic response of colorectal cancer xenograft tumors to sequential treatment with panitumumab and bevacizumab. Neoplasia. 20(7):668-77. doi: 10.1016/j.neo.2018.04.006.  Warbuton C, Dragowska WH, Gelmon K, Chia S, Yan H, Masin D, et al. (2004). Treatment of HER-2/neu overexpressing breast cancer xenograft models with trastuzumab (Herceptin) and gefitinib (ZD1839): drug combination effects on tumor growth, HER-2/neu and epidermal growth factor receptor expression, and viable hypoxic cell fraction. Clin Cancer Res. 10(7):2512-24. doi: 10.1158/1078-0432.ccr-03-0244.  Wang M, Yao L-C, Cheng M, Cai D, Martinek J, Pan C-X. (2018). Humanized mice in studying efficacy and mechanisms of PD-1-targeted cancer immunotherapy. FASEB J. 32(3):1537-49. doi: 10.1096/fj.201700740R.  Zammarchi F, Corbett S, Adams L, Tyrer PC, Kiakos K, Jamghra N, et al. (2018). ADCT-402, a PBD dimer-containing antibody drug conjugate targeting CD19-expressing malignancies. Blood. 131(10):1094-105. doi: 10.1182/blood-2017-10-813493 | | | | | | | | |

| Supplemental Table 2 Intravenous Small Molecule Oncology Drugs | | | | | | | |
| --- | --- | --- | --- | --- | --- | --- | --- |
| **Agent** | **Clinical Dose/ Mechanism/ Indication** | **Clin Dose (mg/kg)** | **Mouse Efficacious Dose (mg/kg)** | **Predicted human Dose by BSA (mg/kg)** | **Xenograft Model (Cell Line)/ Preclinical Dose** | **Preclinical Data Source** | **Ratio** |
| Bendamustine hydrochloride (Bendeka) | 120 mg/m2/ Alkylating/ NHL | 3.24 | 25 | 2.03 | NHL  (Granta 519 cells) 25 mg/kg IV | Ackler et al 2012 | 0.63 |
| Bortezomib (Velcade) | 1.3 mg/m2/ Proteasome inh/  MM | 0.04 | 1 | 0.08 | MM  (RPMI-8226 cells)/  1 mg/kg IV | LeBlanc et al 2002 | 2.31 |
| Busulfan (Busulfex) | 0.8 mg/kg 4xd/ Alkylating/ CML | 3.20 | 20.00 | 1.62 | ALL  (PDX)/ 20 mg/kg IP | Fichtner et al 2003 | 0.51 |
| Cabazetaxil (Jevtana) | 25 mg/m2/ Microtubule inh/  Prostate | 0.68 | 5.6 | 0.45 | Ewings  (TC-71 and SK-ES-1 cells)/  5.6 mg/kg IV | Sidhu et al 2012 | 0.67 |
| Carboplatin (Paraplatin) | 360 mg/m2/ DNA cross-linker/ Ovarian | 9.73 | 75 | 6.08 | Ovarian  (multiple human cell lines)/ 75 mg/kg IP | Brodeur et al 2021 | 0.63 |
| Carfilzomib (Kyprolis) | 27 mg/m2 / Proteasome inh/ MM | 0.73 | 2 | 0.16 | MM  (RPMI-8226 cells)/ 2 mg/kg IV | Park et al 2016 | 0.22 |
| Cisplatin (Platinol) | 20 mg/m2/ Alkylating/ Testicular | 0.54 | 6 | 0.49 | Testicular  (NTera 2 cells)/  6 mg/kg IV | Jørgensen et al 2012 | 0.90 |
| Cladribine (Leustatin) | 0.09 mg/kg/ DNA targeted/  Hairy Cell Leukemia | 0.09 | 30 | 2.43 | Hairy cell Leukemia  (WSU-CLL cells)/  30 mg/kg SC | Mohammad et al 1998 | 27.03 |
| Clofarabine (Clolar) | 52 mg/m2/ DNA targeted/  Lymphoblastic Leukemia | 1.41 | 60 | 4.86 | Lymphoma  (RL Lymphoma)/ 60 mg/kg IP | Bagley et al 2009 | 3.46 |
| Dactinomycin (Cosmegen) | 0.015 mg/kg/ DNA transcription inh/ Rabdomyosarcoma | 0.02 | 0.06 | 0.005 | Colon  (Cx-1 cells)/ 60 ug/kg IV | Sengupta et al 1988 | 0.32 |
| Daunorubicin (Cerubidine) | 45 mg/m2/  Alkylating/  Non-lymphocytic Leukemia | 1.22 | 17 | 1.38 | Breast  (MX-1 cells)/ 17 mg/kg IP | Inoue et al 1983 | 1.13 |
| Docetaxol (Taxotere) | 75 mg/m2 / Microtubule inh/ NSCLC | 2.03 | 23.2 | 1.88 | Lung  (Lewis Lung Carcinoma)/ 23.2 mg/kg IV | Shaik et al 2006 | 0.40 |
| Doxorubicin (Rubex) | 60-75 mg/m2/ Alkylating/  Multiple tumors | 1.82 | 10 | 0.81 | Multiple tumors  (PANC-1 cells)/ 10 mg/kg IV | Peer, Margalit 2004 | 0.44 |
| Eribulin mesylate (Halaven) | 1.4 mg/m2/ Microtubule inh/  Breast | 0.04 | 0.25 | 0.02 | Breast  (MDA-MB-433 cells)/ 0.25 mg/kg IV (ER086526 is eribulin) | Towle et al 2001 | 0.54 |
| Etoposide phosphate (Etopophos) | 35-50 mg/m2 / DNA damage/ SCLC | 1.15 | 40 | 3.24 | Lung  (H291 and LX-1 cells/)/ 40 mg/kg IV | Rose et al 1990 | 2.82 |
| Fludarabine phosphate (Fludara) | 25 mg/m2/ DNA targeted/ CLL | 0.68 | 40 | 3.24 | CLL  (RPMI8226 cells)/ 40 mg/kg IP | Meng et al 2007 | 4.80 |
| Gemcitabine (Gemzar) | 1000 mg/m2/ DNA targeted/  Pancreatic | 27.03 | 120 | 9.73 | Pancreatic  (HS766T, PaCa-2, PANC-1, and BxPC-3 cells)/ 80-160 mg/kg IP | Merriman et al 1996 | 0.36 |
| Ifosfamide (Ifex) | 1200 mg/m2/ Alkylating/ Testicular | 32.43 | 130 | 10.54 | Testicular  (TXF404 and TXF593 cells/PDX)/ 130 mg/kg IP | Berger et al 1990 | 0.33 |
| Irinotecan (Camptosar) | 125 mg/m2/ Topoisomerase inh/ CRC | 3.38 | 60 | 4.86 | CRC  (SW620, COLO205, and HT29 cells)/ 60 mg/kg IV | Hardman et al 1999 | 1.44 |
| Ixebipelone (Ixempra) | 40 mg/m2/ DNA targeted/  Breast | 1.08 | 7.5 | 0.61 | Breast  (BT-474, Pat-14)  6-9 mg/kg IV | Lee et al 2009 | 0.56 |
| Melphalan (Evomela) | 16 mg/m2/ Alkylating/  MM | 0.43 | 3 | 0.24 | MM  (RPMI-8226 cells)/ 3 mg/kg IP | Müller et al 2013 | 0.56 |
| methotrexate (Trexall) | 12 g/m2/ Antimetabolite/  Osteosarcoma | 324.32 | 2400 | 194.59 | Osteosarcoma  (HxOs33 cells)/ 2400 mg/kg IV | Meyer et al 1990 | 0.60 |
| Mitomycin c (Mitozytrex) | 15 mg/m2/ DNA targeted/  Stomach/Pancreatic | 0.41 | 10 | 0.81 | Breast  (MX-1 cells)/ 10 mg/kg IV | Inoue et al 1983 | 2.00 |
| Mitoxantrone (Novantrone) | 12 mg/m2/ DNA targeted/  Acute Non-Lymphocytic Leukemia/Prostate | 0.32 | 3.1 | 0.25 | Breast  (MX-1 cells)/ 3.1 mg/kg IV | Inoue et al 1983 | 0.78 |
| Oxaliplatin (Eloxatin) | 85 mg/m2/ DNA targeted/  CRC (5Fu combination) | 2.30 | 12.5 | 1.01 | Ovarian  (TU-OM-1 cells)/ 12.5 mg/kg IV 5FU combination (25 mg/kg) | Sato et al 2009 | 0.44 |
| Paclitaxel (Taxol) | 135-175 mg/m2/ Antineoplastic/ Ovarian | 4.19 | 30 | 2.43 | Ovarian  (A2780 cells)/ 24-36 mg/kg IV | Lee et al 2001 | 0.58 |
| Pemetrexed disodium (Alimta) | 500 mg/m2/ Folate inh/  NSCLC | 13.51 | 100 | 8.11 | NSCLC  (PC9 cells)/ 100 mg/kg IP | La Monica et al 2016 | 0.60 |
| Pralatrexate (Folotyn) | 30 mg/m2/ Antimetabolite/  T-cell Lymphoma | 0.81 | 15 | 1.22 | Lymphoma  (HH cells)/ 15 mg/kg IP | Marchi et al 2010 | 1.50 |
| Romidepsin (Istodax) | 14 mg/m2/ HDAC inh/  T-cell Lymphoma | 0.38 | 5.6 | 0.45 | Multiple lymphomas  (PDX)/ 5.6 mg/kg IV | Newbold et al 2008 | 1.20 |
| Temsirolimus (Torisel) | 25 mg/  mTORi/ RCC | 0.36 | 10 | 0.81 | RCC  (786-O and Caki-1 cells)/  10 mg/kg IV | Mahalingam et al 2010 | 2.25 |
| Topotecan hydrochloride (Hycamtin) | 1.5 mg/m2/ Topoisomerase inh/  Ovarian | 0.04 | 0.625 | 0.05 | Ovarian  (OVCAR-3 cells)/ 0.625 mg/kg IP | Guichard et al 2001 | 1.25 |
| Trabectedin (Yondelis) | 1.5 mg/m2/ DNA targeted/  Sarcoma | 0.04 | 0.15 | 0.01 | Multiple models/ 0.15 mg/kg IV | Romano et al 2013 | 0.30 |
| Vinblastine (Velban) | 3.7-18.5 mg/m2/ DNA targeted/ Hodgkins Lymphoma/ Testicular | 0.30 | 2.9 | 0.24 | Breast  (MX-1 cells)/ 2.9 mg/kg IV | Inoue et al 1983 | 0.78 |
| Vincristine (Oncovin) | 1.4 mg/m2/ Microtubule inh/  Rhabdomyosarcoma | 0.04 | 1 | 0.08 | Rhabdomyosarcoma (Rh18 cells)/ 1 mg/kg IV | Thompson et al 1999 | 2.14 |
| Vindesine sulphate (Eldisine) | 2-4 mg/m2/ Microtubule inh/  Breast | 0.08 | 1 | 0.08 | Breast  (MX-1 cells)/ 1 mg/kg IV | Inoue et al 1983 | 1.00 |
| BCL: B-cell lymphoma; CLL: chronic lymphocytic leukemia; EGFR: epidermal growth factor receptor; HDAC: histone de-acetylase; HER2: human epidermal growth factor receptor 2; inh: inhibitor; IP: intraperitoneal(ly); IV: intravenous(ly); MM: multiple myeloma; NHL: non-Hodgkin’s lymphoma; NSCLC: non-small cell lung cancer; PD-1: programmed cell death protein 1; PDGFR: platelet-derived growth factor receptor; PDX: patient-derived; SC: subcutaneous(ly); RCC: renal cell carcinoma; SCLC: small cell lung cancer; VEGF: vascular endothelial growth factor receptor.  Ackler S, Mitten MJ, Chen J, Clarin J, Foster K, Jin S, et al. (2012). Navitoclax (ABT-263) and bendamustine ± rituximab induce enhanced killing of non-Hodgkin's lymphoma tumours in vivo. Br J Pharmacol. 167(4):881-91. doi: 10.1111/j.1476-5381.2012.02048.x.  Bagley RG, Roth S, Kurtzberg LS, Rouleau C, Yao M, Crawford J, et al. (2009). Bone marrow CFU-GM and human tumor xenograft efficacy of three antitumor nucleoside analogs. Int J Oncol. 34(5):1329-40.  Berger DP, Fiebig HH, Winterhalter BR, Wallbrecher E, Henss H. (1990). Preclinical phase II study of ifosfamide in human tumour xenografts in vivo. Cancer Chemother Pharmacol. 226 Suppl:S7-S11. doi: 10.1007/BF00685408.  Bissery MC, Guénard D, Guéritte-Voegelein F, Lavelle F. (1991). Experimental antitumor activity of taxotere (RP 56976, NSC 628503), a taxol analogue. Cancer Res. 51(18):4845-52.  Brodeur MN, Simeone K, Leclerc-Deslauniers K, Fleury H, Carmona E, Provencher DM, et al (2021). Carboplatin response in preclinical models for ovarian cancer: comparison of 2D monolayers, spheroids, ex vivo tumors and in vivo models. Nature. 11:18183.  Fichtner I, Becker M, Baumgart J. (2003). Antileukaemic activity of treosulfan in xenografted human acute lymphoblastic leukaemias (ALL). Eur J Cancer. 39(6):801-7. doi: 10.1016/s0959-8049(02)00767-0.  Guichard S, Montazeri A, Chatelut E, Hennebelle I, Bugat R, Canal P. (2001). Schedule-dependent activity of topotecan in OVCAR-3 ovarian carcinoma xenograft: pharmacokinetic and pharmacodynamic evaluation. Clin Cancer Res. 7(10):3222-8.  Hardman WE, Moyer MP, Cameron IL. (1999). Efficacy of treatment of colon, lung and breast human carcinoma xenografts with: doxorubicin, cisplatin, irinotecan or topotecan. Anticancer Res. 19(3B):2269-74.  Inoue K, Fujimoto S, Ogawa M. (1983). Antitumor efficacy of seventeen anticancer drugs in human breast cancer xenograft (MX-1) transplanted in nude mice. Cancer Chemother Pharmacol. 10(3):182-6. doi: 10.1007/BF00255758.  Jørgensen A, Jensen MB, Nielson JE, Juul A, Rajpert-De Meyts E. (2012). Influence of vitamin D on cisplatin sensitivity in testicular germ cell cancer-derived cell lines and in a NTera2 xenograft model. J Steroid Biochem Mol Biol. 136:238-46. doi: 10.1016/j.jsbmb.2012.10.008.  La Monica S, Madeddu D, Tiseo M, Vivo V, Galetti M, Cretella D, et al. (2016). Combination of gefitinib and pemetrexed prevents the acquisition of TKI resistance in NSCLC cell lines carrying EGFR-activating mutation. J Thorac Oncol. 11(7):1051-63. doi: 10.1016/j.jtho.2016.03.006.  LeBlanc R, Catley LP, Hideshima T, Lentzsch S, Mitsiades CS, Mitsiades N, et al. (2002). Proteasome inhibitor PS-341 inhibits human myeloma cell growth in vivo and prolongs survival in a murine model. Cancer Res. 62(17):4996-5000.  Lee FY, Borzilleri R, Fairchild CR, Kim SH, Long BH, Reventos-Suarez C, et al. (2001). BMS-247550: a novel epothilone analog with a mode of action similar to paclitaxel but possessing superior antitumor efficacy. Clin Cancer Res. 7(5):1429-37.  Lee FYF, Smylka R, Johnston K, Menard K, McGlinchey K, Peterson RW. (2009). Preclinical efficacy spectrum and pharmacokinetics of ixabepilone. Cancer Chemother Pharmacol. 63(2):201-12. doi: 10.1007/s00280-008-0727-5.  Mahalingam D, Medina EC, Esquivel 2nd JA, Espitia CM, Smith S, Oberheu K, et al. (2010) Vorinostat enhances the activity of temsirolimus in renal cell carcinoma through suppression of survivin levels. Clin Cancer Res. 2010 Jan 1;16(1):141-53. doi: 10.1158/1078-0432.CCR-09-1385.  Marchi E, Paoluzzi L, Scotto L, Seshan VE, Zain JM, Zinzano PL, et al. (2010). Pralatrexate is synergistic with the proteasome inhibitor bortezomib in in vitro and in vivo models of T-cell lymphoid malignancies. Clin Cancer Res. 16(14):3648-58. doi: 10.1158/1078-0432.CCR-10-0671.  Meng H, Yang C, Ni W, Ding W, Yang X, Qian W. (2007). Antitumor activity of fludarabine against human multiple myeloma in vitro and in vivo. Eur J Haematol. 79(6):486-93. doi: 10.1111/j.1600.0609.2007.00968.x.  Merriman RL, Hertel LW, Schultz RM, Houghton PJ, Houghton JA, Rutherfold PG, et al. (1996). Comparison of the antitumor activity of gemcitabine and ara-C in a panel of human breast, colon, lung and pancreatic xenograft models. Invest New Drugs. 14(3):243-7. doi: 10.1007/BF00194526.  Meyer WH, Loftin SK, Houghton JA, Houghton PJ. (1990). Accumulation, intracellular metabolism, and antitumor activity of high- and low-dose methotrexate in human osteosarcoma xenografts. Cancer Commun. 2(6):219-29. doi: 10.3727/095535490820874407.  Mohammad RM, Katato K, Almatchy VP, Wall N, Liu KZ, Schultz CP, et al. (1998). Sequential treatment of human chronic lymphocytic leukemia with bryostatin 1 followed by 2-chlorodeoxyadenosine: preclinical studies. Clin Cancer Res. 4(2):445-53.  Muller R, Misund K, Holien T, Bachke S, Gilljam KM, Våtsveen TK, et al. (2013). Targeting proliferating cell nuclear antigen and its protein interactions induces apoptosis in multiple myeloma cells. PLoS One. 8(7):e70430. doi: 10.1371/journal.pone.0070430.  Newbold A, Lindemann RK, Cluse LA, Whitecross KF, Dear AE, Johnstone RW. (2008). Characterisation of the novel apoptotic and therapeutic activities of the histone deacetylase inhibitor romidepsin. Mol Cancer Ther. 7(5):1066-79. doi: 10.1158/1535-7163.MCT-07-2256.  Park J, Park E, Jung C-K, Kang S-W, Kim BG, Jung Y, et al. (2016). Oral proteasome inhibitor with strong preclinical efficacy in myeloma models. BMC Cancer. 16:247. Doi: 10.1186/s12885-016-2285-2.  Peer D, Margalit. (2004). Tumor-targeted hyaluronan nanoliposomes increase the antitumor activity of liposomal doxorubicin in syngeneic and human xenograft mouse tumor models. Neoplasia. 6(4):343-53. doi:10.1593/neo.03460.  Romano M, Frapolli R, Zangarini M, Bello E, Porcu L, Galmarini CM, et al. (2013). Comparison of in vitro and in vivo biological effects of trabectedin, lurbinectedin (PM01183) and Zalypsis (PM00104). Int J Cancer. 133(9):2024-33. doi: 10.1002/ijc.28213.  Rose WC, Basler GA, Trail PA, Saulnier M, Crosswell AR, Casazza AM. (1990). Preclinical antitumor activity of a soluble etoposide analog, BMY-40481-30. Invest New Drugs. 8 Suupl 1:S25-S32. doi: 10.1007/BF00171981.  Sato S, Itamochi H, Kigawa J, Oishi T, Shimada M, Sato S. (2009). Combination chemotherapy of oxaliplatin and 5-fluorouracil may be an effective regimen for mucinous adenocarcinoma of the ovary: a potential treatment strategy. Cancer Sci. 100(3):546-51. doi: 10.1111/j.1349-7006.2008.01065.x.  Sengupta SK, Kogan Y, Kelly C, Szabo J. (1988). New actinomycin D analogs as superior chemotherapeutic agents against primary and advanced colon tumors and colon xenografts in nude mice. J Med Chem. 31(4) :768-74. doi: 10.1021/jm00399a014.  Shaik MS, Chatterjee A, Jackson T and Mandip S. (2006) Enhancement of antitumor activity of docetaxel by celecoxib in lung tumors. Int J Cancer 118:396‑404. doi: 10.1002/ijc.21325.  Sidhu SS, Malfilatre M, Janvier C, Boulcourt-Sambou E, Vincent L, Calvet L, et al. (2012). Abstract 2775: antitumor activity of cabazitaxel in pediatric tumor xenografts. Cancer Res. 72(8):Supp 1. doi: 10.1158/1538-7445.AM2012-2775.  Thompson J, George EO, Poquette CA, Cheshire PJ, Richmond LB, de Graaf SS, et al. (1999). Synergy of topotecan in combination with vincristine for treatment of pediatric solid tumor xenografts. Clin Cancer Res. 5(11):3617-31.  Towle MJ, Salvato KA, Budrow J, Wels BF, Kuznetsov G, Aalfs KK, et al. (2001). In vitro and in vivo anticancer activities of synthetic macrocyclic ketone analogues of halichondrin B. Cancer Res. 61(3):1013-21. | | | | | | | |

| Supplemental Table 3 Oral Small Molecule Oncology Drugs | | | | | | | |
| --- | --- | --- | --- | --- | --- | --- | --- |
| **Agent** | **Clinical Dose/ Mechanism/ Indication** | **Clin Dose (mg/kg)** | **Mouse Efficacious Dose (mg/kg)** | **Predicted human Dose by BSA (mg/kg)** | **Xenograft Model (Cell Line)/ Preclinical Dose** | **Preclinical Data Source** | **Ratio** |
| Afatinib (Gilotrif) | 40 mg/ EGFRi/ NSCLC | 0.57 | 10 | 0.81 | NSCLC  (RPC-9 or H1975 cells)/ 10 mg/kg PO | Ninomiya et al 2013 | 1.42 |
| Alectinib (Alecensa) | 600 mg BID/ ALK inh/ NSCLC | 17.1 | 60 | 4.86 | NSCLC  (NCI-H2228 cells)/ 60 mg/kg PO | Kodama et al 2014 | 0.28 |
| Anastrozole (Arimidex) | 1 mg/ Aromatase inh/ Breast | 0.01 | 0.2 | 0.02 | Breast  (MCF-7)/  5 ug/mouse SC | Brodie et al 1999 | 1.16 |
| Avapritinib (Ayvakit) | 300 mg/ PDGFR inh/ GIST | 4.28 | 60 | 4.86 | GIST  (UZLX-GIST2B PDX)/ 60 mg/kg PO | Gebreyohannes et al 2019 | 1.14 |
| Axitinib (Inlyta) | 5mg BID/ VEGFR inh/ RCC | 0.14 | 60 | 4.86 | RCC  (SN12c GFP cells) 30 mg/kg BID PO | Hu-Lowe et al 2008 | 36.0 |
| Bosutinib (Bosulif) | 500 mg/ BCR-ABL inh/ CML | 7.14 | 100 | 8.11 | CML  (K562 cells)/ 100 mg/kg PO | Golas et al 2003 | 1.14 |
| Brigatinib (Alunbrig) | 180 mg/ ALK inh/ ALK+ NSCLC | 2.57 | 25 | 2.03 | NSCLC  (Karpas-299, H2228 cells)/ 25 mg/kg PO | Zhang et al 2016 | 0.79 |
| Cabozantinib (Cometriq) | 140 mg/  MET/RET/VEGFR inh/  Thyroid | 2 | 60 | 4.86 | Thyroid  (TT cells/non-PDX)/  60 mg/kg PO | Bentzien et al 2013 | 2.43 |
| Capecitabine (Xeloda) | 1250 mg/m2 BID/ DNA targeted/  CRC | 67.57 | 467 | 37.86 | CRC  (HT29 CRC cells)/ 467 mg/kg PO | Kolinsky et al 2009 | 0.56 |
| Capmatinib (Tabrecta) | 400 mg BID/ MET inh/ NSCLC | 11.4 | 20 | 1.62 | MET+ NSCLC  (PDX)/ 10mg/kg BID PO | Baltschukat et al 2019 | 0.14 |
| Cobimetinib (cotellic) | 60 mg/ BRAF inh/ Melanoma | 0.86 | 5 | 0.41 | Melanoma  (A375.X1 BRAF^V600E^)/  5 mg/kg PO | Hoeflich et al 2012 | 0.47 |
| Crizotinib (Xalkori) | 250 mg BID/ ALK/ROS1inh/ NSCLC | 7.14 | 100 | 8.11 | NSCLC  (EML4-ALK dependent NSCLC)/ 100 mg/kg PO | Zhang et al 2011 | 1.14 |
| Cyclophosphamide (Cytoxan) | 1-5 mg/kg/ Alkylating/ multiple | 3.00 | 30 | 2.43 | Multiple 20-40 mg/kg PO | Man et al 2002 | 0.81 |
| Dabrafenib (Tafinlar) | 150 mg BID/ BRAF inh/ Melanoma | 4.29 | 100 | 8.11 | CRC  (Colo 205)/ 100 mg/kg PO | King et al 2013 | 1.89 |
| Dacomitinib (Vizimpro) | 45 mg/ EGFR inh/ NSCLC | 0.64 | 15 | 1.22 | NSCLC  (SKOV3 and H125 cells)/ 15 mg/kg PO | Gonzales et al 2008 | 1.90 |
| Dasatinib (Sprycel) | 100 mg/ BCR-ABL inh/ CML | 1.43 | 30 | 2.43 | CML  (K562 cells)/ 15 mg/kg BID PO | Wild et al 2004 | 1.70 |
| Everolimus (Afinitor) | 10 mg/ mTOR inh/  RCC | 0.14 | 2.00 | 0.16 | Hepatic  (Caki-1 cells)/ 2 mg/kg PO | Zou et al 2017 | 1.16 |
| Erlotinib (Tarceva) | 150 mg/ EGFR inh/ NSCLC | 2.14 | 50 | 4.05 | NSCLC  (NCI-H322M cells[NCI-H522 or Calu-3 cells)/ 50 mg/kg PO | Friess et al 2006 | 1.89 |
| Hexamethylmelamine (Altretamine) | 260 mg/m2 / Cytotoxic/ Ovarian | 7.03 | 200 | 16.22 | Breast  (MX-1 cells)/  200 mg/kg IP | Inoue et al 1983 | 2.31 |
| Imatinib mesylate (Gleevec) | 400 mg/ BCR-ABL inh/ GIST | 5.71 | 100 | 8.11 | GIST  (Human GIST AHAX)/ 100 mg/kg PO | Revheim et al 2013 | 1.42 |
| Gefitinb (Iressa) | 250 mg/ EGFRi/  NSCLC | 3.57 | 50.00 | 4.05 | NSCLC  (A431 cells [A549 or Du145 cells])/ 50 mg/kg PO | Wakeling et al 2002 | 1.13 |
| Gilteritinib (Xospada) | 120 mg/ FLT3 inh/ AML | 1.71 | 6 | 0.49 | AML  (MV4-11 cells)/ 6 mg/kg PO | Mori et al 2017 | 0.28 |
| Ibrutinib (Imbruvica) | 420 mg/ BTK inh/  CLL | 6 | 25 | 2.03 | CLL  (TCL1 leukemia cells)/ 25 mg/kg PO | Ponader et al 2012 | 0.34 |
| Ixazomib (Ninlaro) | 4 mg/ Proteasome inh/ MM | 0.057 | 5 | 0.41 | MM  (MM.1S cells)/ 5 mg/kg PO | Park et al 2016 | 7.11 |
| Lapatinib (Tykerb) | 1250 mg/ EGFR/HER2i/ Breast | 17.86 | 200 | 16.22 | Breast  (BT474 cells)/ 100 mg/kg PO BID | Gaul et al 2003 | 0.91 |
| Larotrectinib (Vitrakvi) | 100 mg BID/ TRK inh/ Pediatric tumors | 2.86 | 100 | 8.11 | TRK+ mammary  (NTRK3 PDX cells)/ 100 mg/kg PO | Somwar et al 2020 | 2.84 |
| Lenvatinib (Lenvima) | 24 mg/ VEGFR inh/ Thyroid | 0.34 | 10 | 0.81 | Thyroid  (Multiple)/ 10 mg/kg PO | Tohyama et al 2014 | 2.38 |
| Lorlatinib (Lorbrena) | 100 mg/ ALK inh/ ALK+ NSCLC | 1.43 | 20 | 1.62 | NSCLC  (H3122-EML4-ALK and 3T3-EML4-ALK cells)/ 20-25 mg/kg PO | Zou et al 2015 | 1.13 |
| Mercaptopurine (Purinethol) | 1.5-2.5 mg/kg/ Unclear mechanism/ ALL | 2.00 | 100 | 8.11 | ALL  (Rosa26+ cells)/ 50 mg/kg BID IP | Tzoneva et al 2018 | 4.05 |
| Mitotane (Lysodren) | 2-6 g/day (assumes dose of 4 g)/ Cytotoxic/ Adrenal Cortical Carcinoma | 57.1 | 440 | 35.68 | Adrenal Cell Carcinoma  (H295R cells)/ 440 mg/kg PO | Doghman, Lalli 2013 | 0.62 |
| Nilotinib (Tasigna) | 400 mg BID/ BCR-ABL inh/ CML | 11.40 | 100 | 8.11 | CEL  (EOL-1 cells)/ 100 mg/kg PO | Wicklein et al 2012 | 0.71 |
| Niraparib (Zejula) | 300 mg/ PARP inh/ Ovarian | 4.29 | 100 | 8.11 | Ovarian  (PDX)/ 100 mg/kg PO | AlHilli et al 2016 | 1.89 |
| Olaparib (Lynparza) | 300 mg BID/ PARP inh/ Ovarian | 8.57 | 75 | 6.08 | Ovarian  (BRCA PDX cells)/ 75 mg/kg PO | Sun et al 2018 | 0.71 |
| Osimertinib (Tagrisso) | 80 mg/ EGFR inh/ NSCLC | 1.14 | 5 | 0.41 | NSCLC  (H3255 and PC-9VanR cells)/ 5 mg/kg PO | Cross et al 2014 | 0.36 |
| Palbociclib (Ibrance) | 125 mg/ CDK4/6 / Breast | 1.79 | 100 | 8.11 | Breast  (H1975 cells)/ 100 mg/kg/PO | Bisi et al 2017 | 4.53 |
| Panobinostat (Farydak) | 20 mg/ HDAC inh/ MM | 0.29 | 7.5 | 0.61 | Lymphoma  (HH human CTCL cells)/ 7.5 mg/kg PO | Shao et al 2010 | 2.10 |
| Pazopanib (Votrient) | 800 mg/ VEGFR1 inh/ RCC | 11.40 | 100 | 8.11 | RCC  (Caki-1 cells)/ 100 mg/kg PO | Kumar et al 2007 | 0.71 |
| Pemigatinib (Pemazyre) | 13.5 mg/ FGFR2 inh/ Cholangiocarcinoma | 0.19 | 1 | 0.08 | Cholangiosarcoma  (PDX)/ 1 mg/kg PO | Liu et al 2020 | 0.43 |
| Ponatinib (Iclusig) | 45 mg/  BCR-ABL in/ CML | 0.64 | 10 | 0.81 | Resistant AML  (MV4-11 cells)/  10 mg/kg PO | Gozgit et al 2011 | 1.27 |
| Pralsetinib (Gavreto) | 400 mg/ RET inh/ NSCLC | 5.71 | 60 | 4.86 | NSCLC (PDX)/ 30 mg/kg BID PO | Subbiah et al 2018 | 0.85 |
| Regorafenib (Stivarga) | 160 mg/ VEGFR1 inh / CRC | 2.28 | 10 | 0.81 | CRC  (PDX)/ 10 mg/kg PO | Schmieder et al 2014 | 0.36 |
| Ripretinib (Qinlock) | 150 mg/ KIT and PDGFR inh/ GIST | 2.14 | 25 | 2.03 | GIST (GIST T1)/ In chow 25 mg/kg | Gupta et al 2021 | 0.95 |
| Selinexor (Xpovio) | 60-80 mg / Nuclear export inh / MM and DLBCL | 1.00 | 15 | 1.22 | multiple sarcoma  (PDX)/ 15 mg/kg PO | Nakayama et al 2016 | 1.22 |
| Sonidegib (Odomzo) | 200 mg/ HH inh/ BCC | 2.86 | 60 | 4.86 | Melanoma (A375 cells)/ 60 mg/kg PO | O’Reilly et al 2013 | 1.70 |
| Sorafenib (Nexavar) | 200 mg BID/ BRAF inh/ HCC | 5.71 | 100 | 8.11 | HCC  (PLC/PFR/5 cells)/ 100 mg/kg PO | Liu et al 2006 | 1.42 |
| Sunitinib malate (Sutent) | 50 mg/ VEGFR inh/ RCC | 0.71 | 20 | 1.62 | RCC  (786-O cells)/ 20 mg/kg PO | Zhou et al 2016 | 2.28 |
| Talazoparib (Talzenna) | 1 mg/ PARP inh/ Breast | 0.014 | 0.33 | 0.03 | Breast  (MX-1 cells)/ 0.33 mg/kg PO | Shen et al 2013 | 1.91 |
| Tazemetostat (Tazverik) | 800 mg BID/ Methyltransferase inh/ follicular lymphoma | 22.9 | 500 | 40.54 | Sarcoma (PDX)/ 250 mg/kg BID PO | Kawano et al 2016 | 1.77 |
| Temozolomide (Temodar) | 150 mg/m2/ MGMT inh/ Glio or astrocytoma | 4.05 | 50 | 4.05 | Glioblastoma  (GBM12 cells)/ 50 mg/kg PO | Kitange et al 2009 | 1.00 |
| Tepotinib (Tepmetko) | 450 mg/ MET inh/ NSCLC | 6.43 | 100 | 8.11 | MET+ NSCLC  (PDX)/ 100 mg/kg PO | Friese-Hamim et al 2017 | 1.26 |
| Trametinib (Mekinist) | 2 mg/ BRAF inh/ Melanoma/ NSCLC | 0.029 | 0.3 | 0.02 | CRC  (PDX)/ 0.3 mg/kg PO | Walters et al 2013 | 0.84 |
| Tucatinib (Tukysa) | 300 BID/ HER2 inh/ Breast | 8.57 | 100 | 8.11 | Breast  (PDX)/ 50 mg/kg PO | Kulukian et al 2020 | 0.95 |
| Vandetanib (Caprelsa) | 300 mg/ EGFR/VEGFR inh/ Thyroid | 4.29 | 50 | 4.05 | Thyroid  (MTC cells)/ 50 mg/kg PO | Brassard, Rondeau 2012 | 0.95 |
| Vemurafenib (Zelboraf) | 960 mg BID/ BRAF inh/ Melanoma | 27.43 | 100 | 8.11 | Melanoma  (PDX)/ 50mg/kg PO BID | Monsma et al 2015 | 0.30 |
| Venetoclax (Venclexta) | 400 mg/ BCL-2 inh/ CLL | 5.71 | 100 | 8.11 | ALL  (PDX)/ 100 mg/kg PO | Khaw et al 2016 | 1.42 |
| Vismodegib (Erivedge) | 150 mg/ HH inh/ BCC | 2.14 | 25 | 2.03 | Medulloblastoma  (PDX)/ 25 mg/kg PO | Wong et al 2011 | 0.95 |
| ALK: anaplastic lymphoma kinase; ALL: acute lymphocytic leukemia; AML: acute myeloid leukemia; BCR-ABL: fusion of BCR and ABL genes linked to certain leukemias; BCC: basal cell carcinoma; BCL: B-cell lymphoma; BID: twice daily; CEL: chronic eosinophilic leukemia; CLL: chronic lymphocytic leukemia; CML: chronic myeloid leukemia; CRC: colorectal cancer; DLBCL: diffuse large B-cell lymphoma; EGFR: epidermal growth factor receptor; FGFR2: fibroblast growth factor receptor 2; GIST: gastrointestinal stromal tumor; HCC: hepatocellular carcinoma; HDAC: histone de-acetylase; HER2: human epidermal growth factor receptor 2; inh: inhibitor; IP: intraperitoneal(ly); IV: intravenous(ly); MET: mesenchymal epithelial transition factor; MGMT: methylguanine DNA methyltransferase; NSCLC: non-small cell lung cancer; PARP: polyadenosine diphosphate ribose polymerase; PDGFR: platelet-derived growth factor receptor PDX: patient-derived; PO: oral(ly); RCC: renal cell carcinoma; RET: rearranged during transfection; SC: subcutaneous(ly); VEGFR: vascular endothelial growth factor receptor.  AlHilli MM, Becker MA, Weroha SJ, Flatten KS, Hurley RM, Harrell MI, et al. (2016). In vivo anti-tumor activity of the PARP inhibitor niraparib in homologous recombination deficient and proficient ovarian carcinoma. Gynecol Oncol. 143(2):379-88. doi: 10.1016/j.ygyno.2016.08.328.  Baltschukat S, Engstler BS, Huang A, Hao H-X, Tam A, Wang HQ, et al. (2019). Capmatinib (INC280) is active against models of non-small cell lung cancer types with defined mechanisms of MET activiation. Clin Cancer Res. 25(10):3164-75. doi: 10.1158/1078-0432.CCR-18-2814.  Bentzien F, Zuzow M, Heald N, Gibson A, Shi Y, Goon L, et al (2013). In vitro and in vivo activity of cabozantinib (XL184) an inhibitor of RET, MET, and VEGFR2, in a model of medullary thyroid cancer. Thyroid. 23(12):1569-77. doi: 10.1089/thy.2013.0137.  Bisi JE, Sorrentino JA, Jordan JL, Darr DD, Roberts PJ, Tavares FX, et al. (2017). Preclinical development of G1T38: a novel, potent and selective inhibitor of cyclin dependent kinases 4/6 for use as an oral antineoplastic in patients with CDK4/6 sensitive tumors. Oncotarget. 8(26):42343-58. doi: 10.18632/oncotarget.16216.  Brassard M, Rondeau G. (2012). Role of vandetanib in the management of medullary thyroid cancer. Biologics. 6:59-66. doi: 10.2147/BTT.S24220.  Brodie A, Lu Q, Liu Y, Long B. (1999). Aromatase inhibitors and their antitumor effects model systems. Endocr Relat Cancer. 6(2):205-210. doi: 10.1677/erc.0.0060205  Cross DAE, Ashton SE, Ghiorghiu S, Eberlein C, Nebhan CA, Spitzler PJ, et al. (2014). AZD9291, an irreversible EGFR TKI, overcomes T790M-mediated resistance to EGFR inhibitors in lung cancer. Cancer Discov. 4(9):1046-61. doi: 10.1158/2159-8290.CD-14-0337.  Doghman M, Lalli E. (2013). Lack of long-lasting effects of mitotane adjuvant therapy in a mouse xenograft model of adrenocortical carcinoma. Mol Cell Endocrinol. 381(1-2):66-9. doi: 10.1016/j.mce.2013.07.023.  Friese-Hamim M, Bladt F, Locatelli G, Stammberger U, Blaukat A. (2017). The selective c-MET inhibitor tepotinib can overcome epidermal growth factor receptor inhibitor resistance mediated by aberrant c-Met activation in NSCLC models. Am J Cancer Res. 7(4):962-72.  Friess T, Scheuer W, Hasmann M. (2006). Erlotinib antitumor activity in non-small cell lung cancer models is independent of HER1 and HER 2 overexpression. Anticancer Res. 26(5A):3505-12.  Gaul MD, Guo Y, Affleck K, Cockerill GS, Gilmer TM, Griffin RJ, et al. (2003). Discovery and biological evaluation of potent dual ErbB-2/EGFR tyrosine kinase inhibitors: 6-thiazolylquinazolines. Bioorg Med Chem Lett. 13(4):637-40. doi: 10.1016/s0960-894x(02)01047-8.  Gebreyohannes YK, Wozniak A, Zhai M-E, Wellens J, Cornillie J, Vanleeuw U, et al. (2019). Robust activity of avapritinib, potent and highly selective inhibitor of mutated KIT, in patient-derived xenograft models of gastrointestinal stromal tumors. Clin Cancer Res. 25(2):609-18. doi: 10.1158/1078-0432.CCR-18-1858.  Golas JM, Arndt K, Etienne C, Lucas J, Nardin D, Gibbons J, et al. (2003). SKI-606, a 4-anilino-3-quinolinecarbonitrile dual inhibitor or Src and Abl kinases, is a potent antiproliferative agent against chronic myelogenous leukemia cells in culture and causes regression of K562 xenografts in nude mice. Cancer Res. 63(2):375-81.  Gonzales AJ, Hook KE, Althaus IW, Ellis PA, Trachet E, Delaney AM, et al. (2008). Antitumor activity and pharmacokinetic properties of PF-00299804, a second-generation irreversible pan-erbB receptor tyrosine kinase inhibitor. Mol Cancer Ther. 7(7):1880-9. doi: 10.1158/1535-7163.MCT-07-2232.  Gozgit JM, Wong MJ, Wardwell S, Tyner JW, Loriaux MM, Mohemmad QK, et al. (2011). Potent activity of ponatinib (AP24534) in models of FLT3-driven acute myeloid leukemia and other hematologic malignancies. Mol Cancer Ther. 10(6):1028-35. doi: 10.1158/1535-7163.MCT-10-1044.  Gupta A, Singh J, García-Valverde A, Serrano C, Flynn DL, Smith BD. (2021). Ripretinib and MEK inhibitors synergize to induce apoptosis in preclinical models of GIST and systemic mastocytosis. Mol Cancer Ther. 20(7):1234-45. doi: 10.1158/1535-7163.MCT-20-0824.  Hoeflich KP, Merchant M, Orr C, Chan J, Otter DD, Berry L, et al. (2012). Intermittent administration of MEK inhibitor GDC-0973 plus PI3K inhibitor GDC-0941 triggers robust apoptosis and tumor growth inhibition. Cancer Res. 72(1):210-9. doi: 10.1158/0008-5472.CAN-11-1515.  Hu-Lowe DD, Zou HY, Grazzini ML, Hallin ME, Wickman GR, Amundson K, et al. (2008). Nonclinical antiangiogenesis and antitumor activities of axitinib (AG-013736), an oral, potent, and selective inhibitor of vascular endothelial growth factor receptor tyrosine kinases 1, 2, 3. Clin Cancer Res. 14(22):7272-83. doi: 10.1158/1078-0432.CCR-08-0652.  Inoue K, Fujimoto S, Ogawa M. (1983). Antitumor efficacy of seventeen anticancer drugs in human brease cancer xenograft (MX-1) transplanted in nude mice. Cancer Chemother Pharmacol. 10(3):182-6. doi: 10.1007/BF00255758.  Kawano S, Grassian AR, Tsuda M, Knutson SK, Warholic NM, Kuznetsov G, et al. (2016). Preclinical evidence of anti-tumor activity induced by EZH2 inhibition in human models of synovial sarcoma. PLoS One. 11(7):e0158888. doi: 10.1371/journal.pone.0158888.  Khaw SL, Suryani S, Evans K, Richmond J, Robbins A, Kurmasheva RT, et al. (2016). Venetoclax responses of pediatric ALL xenografts reveal sensitivity of MLL-rearranged leukemia. Blood. 128(10):1382-95. doi: 10.1182/blood-2016-03-707414.  King AJ, Arnone MR, Bleam MR, Moss KG, Yang J, Fedorowicz KE, et al. (2013). Dabrafenib; preclinical characterization, increased efficacy when combined with trametinib, while BRAF/MEK tool combination reduced skin lesions. PLoS One. 8(7):e67583. doi: 10.1371/journal.pone.0067583.  Kitange GJ, Carlson BL, Schroeder MA, Grogan PT, Lamont JD, Decker PA, et al. (2009). Induction of MGMT expression is associated with temozolomide resistance in glioblastoma xenografts. 11(3). 281-91. doi: 10.1215/15228517-2008-090.  Kodama T, Tsukaguchi T, Yoshida M, Kondoh S, Sakamoto H. (2014). Selective ALK inhibitor alectinib with potent antitumor activity in models of crizotinib. Cancer Lett. 351(2):215-21. doi: 10.1016/j.canlet.2014.05.020.  Kolinsky K, Zhang Y-E, Dugan U, Heimbrook D, Packman, Higgins B. (2009). Novel regimens of capecitabine alone and combined with irinotecan and bevacizumab in colorectal cancer xenografts. Anticancer Res. 29(1):91-8.  Kulukian A, Lee P, Taylor J, Rosler R, de Vries P, Watson D, et al. (2020). Preclinical activity of HER2-selective tyrosine kinase inhibitor tucatinib as a single agent or in combination with trastuzumab or docetaxel in solid tumor models. Mol Cancer Ther. 19(4):976-87. doi: 10.1158/1535-7163.MCT-19-0873.  Kumar R, Knick VB, Rudolph SK, Johnson JH, Crosby RM, Crouthamel M-C, et al. (2007). Pharmacokinetic-pharmacodynamic correlation from mouse to human with pazopanib, a multikinase angiogenesis inhibitor with potent antitumor and antiangiogenic activity. Mol Cancer Ther. 6(7):2012-21. doi: 10.1158/1535-7163.MCT-07-0193.  Liu L, Cao Y, Chen C, Zhang X, McNabola A, Wilkie D, et al. (2006). Sorafenib blocks the RAF/MEK/ERK pathway, inhibits tumor angiogenesis, and induces tumor cell apoptosis in hepatocellular carcinoma model PLC/PRF/5. 66(24):11851-8. doi: 10.1158/0008-5472.CAN-06-1377.  Liu PCC, Koblish H, Wu L, Bowman K, Diamond S, Di Matteo D, et al. (2020). INCB054828 (pemigatinib), a potent and selective inhibitor of fibroblast growth factor receptors 1, 2, and 3, displays activity against genetically defined tumor models. PLoS One. 15(4):e0231877. doi: 10.1371/journal.pone.0231877.  Man S, Bocci G, Francia G, Green SK, Jothy S, Hanahan D, et al. (2002). Antitumor effects in mice of low-dose (metronomic) cyclophosphamide administered continuously through the drinking water. Cancer Res. 62(10):2731-5.  Monsma DJ, Cherba DM, Eugster EE, Dylewski DL, Davidson PT, Peterson CA, et al. (2015). Melanoma patient derived xenografts acquire distinct vemurafenib resistance mechanisms. Am J Cancer Res. 5(4):1507-18.  Mori M, Kaneko N, Ueno Y, Yamada M, Tanaka R, Saito R, et al. (2017). Gilteritinib a FLT3/AXL inhibitor, shows antileukemic activity in mouse models of FLT3 mutated acute myeloid leukemia. Invest New Drugs. 35(5):556-65. doi: 10.1007/s10637-017-0470-z.  Nakayama R, Zhang Y-X, Czaplinski JT, Anatone AJ, Sicinska ET, Fletcher JA, et al. (2016). Preclinical activity of selinexor, an inhibitor of XPO1, in sarcoma. Oncotarget. 7(13):16581-92. doi: 10.18632/oncotarget.7667.  Ninomiya T, Takigawa N, Ichihara E, Ochi N, Murakami T, Honda Y, et al. (2013). Afatinib prolongs survival compared with gefitinib in an epidermal growth factor receptor-driven lung cancer model. Mol Cancer Ther. 12(5):589-97. doi: 10.1158/1535-7163.MCT-12-0885.  O’Reilly K, Vega-Saenz de Miera E, Segura MF, Friedman E, Poliseno L, Han SW, et al. (2013). Hedgehog pathway blockade inhibits melanoma cell growth in vitro and in vivo. Pharmaceuticals (Basel). 6(11):1429-50. doi: 10.3390/ph6111429.  Park J, Park E, Jung C-K, Kang S-W Kim BG, Jung Y, et al. (2016). Oral proteasome inhibitor with strong preclinical efficacy in myeloma models. BMC Cancer. 16:247. doi: 10.1186/s12885-016-2285-2.  Ponader S, Chen S-S, Buggy JJ, Balakrishnan K, Gandhi V, Wierda WG, et al. (2012). The Bruton tyrosine kinase inhibitor PCI-32765 thwarts chronic lymphocytic leukemia cell survival and tissue homing in vitro and in vivo. Blood. 119(5):1182-9. doi: 10.1182/blood-2011-10-386417. AND Herman SEM, Sun X, McAuley EM, Hsieh MM, Pittaluga S, Raffeld M, et al. (2013). Modeling tumor-host interactions of chronic lymphocytic leukemia in xenografted mice to study tumor biology and evaluate targeted therapy. Leukemia. 27(12):2311-21. doi: 10.1038/leu.2013.131.  Revheim M-E, Kristian A, Malinen E, Bruland ØS, Berner J-M, Holm R, et al. (2013). Intermittent and continuous imatinib in a human GIST xenograft model carrying KIT exon 17 resistance mutation D816H. 52(4):776-82. doi:10.3109/0284186X.2013.770920.  Schmieder R, Hoffman J, Becker M, Bhargava A, Müller T, Kahmann N, et al. (2014). Regorafenib (BAY 73-4506): antitumor and antimetastatic activities in preclinical models of colorectal cancer. 135(6):1487-96. doi: 10.1002/ijc.28669.  Shao W, Growney JD, Feng Y, O'Conner F, Pu M, Zhu W, et al. (2010). Activity of deacetylase inhibitor panobinostat (LBH589) in cutaneous T-cell lymphoma models: defining molecular mechanisms of resistance. Int J Cancner. 127(9):2199-208. doi: 10.1002/ijc.25218.  Shen Y, Rehman FL, Feng Y, Boshuizen J, Bajrami I, Elliott R, et al. (2013). BMN 673, a novel and highly potent PARP1/2 inhibitor for the treatment of human cancers with DNA repair deficiency. Clin Cancer Res. 19(18):5003-15. doi: 10.1158/1078-0432.CCR-13-1391.  Somwar R, Hofmann NE, Smith B, Odintsov I, Vojnic M, Linkov I, et al. (2020). NTRK kinase domain mutations in cancer variably impact sensitivity to type I and type II inhibitors. Commun Biol. 3(1):776. doi: 10.1038/s42003-020-01508-w.  Subbiah V, Gainor JF, Rahal R, Brubaker JD, Kim JL, Maynard M, et al. (2018). Precision targeted therapy with BLU-667 for RET-driven cancers. Cancer Discov. 8(7):836-49. doi: 10.1158/2159-8290.CD-18-0338.  Sun K, Mikule K, Wang Z, Poon G, Vaidyanathan A, Smith G, et al. (2018). A comparative pharmacokinetic study of PARP inhibitors demonstrates favorable properties for niraparib efficacy in preclinical tumor models. Oncotarget. 9(98):37080-96. doi: 10.18632/oncotarget.26354.  Tohyama O, Matsui J, Kodama K, Hata-Sugi N, Kimura T, Okamoto K, et al. (2014). Antitumor activity of lenvatinib (e7080): an angiogenesis inhibitor that targets multiple receptor tyrosine kinases in preclinical human thyroid cancer models. J Thyroid Res. 2014:638747. doi: 10.1155/2014/638747.  Tzoneva G, Dieck CL, Oshima K, Ambesi-Impiombato A, Sánchez-Martín M, Madubata CJ, et al. (2018). Clonal evolution mechanisms in NT5C2 mutant-relapsed acute lymphoblastic leukemia. Nature. 553(7689):511-4. doi: 10.1038/nature25186.  Wakeling AE, Guy SP, Woodburn JR, Ashton SE, Curry BJ, Barker AJ, et al. (2002). ZD1839 (Iressa): an orally active inhibitor of epidermal growth factor signaling with potential for cancer therapy. 62(20):5749-54.  Walters DM, Lindberg JM, Adair SJ, Newhook TE, Cowan CR, Stokes JB, et al (2013). Inhibition of the growth of patient-derived pancreatic cancer xenografts with the MEK inhibitor trametinib is augmented by combined treatment with the epidermal growth factor receptor/HER2 inhibitor lapatinib. Neoplasia. 15(2):143-55. doi: 10.1593/neo.121712  Wicklein D, Leal NR, Salamon J, Thamer M, Herrmann H, Valent P, et al. (2012). Nilotinib and imatinib are comparably effective in reducing growth of human eosinophil leukemia cells in a newly established xenograft model. PLoS One. 7(2):e30567. doi: 10.1371/journal.pone.0030567.  Wild R, Castaneda S, Flefleh C, Fager K, Inigo I, Kan D, et al. (2004). BMS-354825, a dual SRC/ABL kinase inhibitor, displays potent anti-tumor activity in a model of intracranial CML growth. Blood.104(11):1988.  Wong H, Alicke B, West KA, Pacheco P, La H, Januario T, et al. (2011). Pharmacokinetic-pharmacodynamic analysis of vismodegib in preclinical models of mutational and ligand-dependent hedgehog pathway activation. Clin Cancer Res. 17(14):4682-92. doi: 10.1158/1078-0432.CCR-11-0975.  Zhang S, Wang F, Keats J, Zhu X, Ning Y, Wardwell SD, et al. (2011). Crizotinib-resistant mutants of EML4-ALK identified through an accelerated mutagenesis screen. Chem Biol Drug Des. 78(6):999-1005. doi: 10.1111/j.1747-0285.2011.01239.x.  Zhang S. Anjum R, Squillace R, Nadworny S, Zhou T, Keats J, et al. (2016). The potent ALK inhibitor brigatinib (AP26113) overcomes mechanisms of resistance to first- and second-generation ALK inhibitors in preclinical models. Clin Cancer Res. 22(22):5527-38. doi: 10.1158/1078-0432.CCR-16-0569.  Zhou L, Liu X-D, Sun M, Zhang X, German P, Bai S, et al. (2016). Targeting MET and AXL overcomes resistance to sunitinib therapy in renal cell carcinoma. Oncogene. 35(21):2687-97. doi: 10.1038/onc.2015.343.  Zou HY, Friboulet L, Kodack DP, Engstrom LD, Li Q, West M, et al. (2015). PF-06463922, an ALK/ROS1 inhibitor, overcomes resistance to first and second generation ALK inhibitors in preclinical models. Cancer Cell. 28(1):70-81. doi: 10.1016/j.ccell.2015.05.010.  Zou Y, Wang J, Leng X, Huang J, Xue W, Zhang J, et al. (2017). The selective MEK1 inhibitor selumetinib enhances the antitumor activity of evorolimus against renal cell carcinoma in vitro and in vivo. Oncotarget. 8(13):20825-33. doi: 10.18632/oncotarget.15346. | | | | | | | |

| Supplemental Table 4 Immune/Endocrine Oncology Drugs | | | | | | | |
| --- | --- | --- | --- | --- | --- | --- | --- |
| **Agent** | **Clinical Dose/ Mechanism/ Indication** | **Clin Dose (mg/kg)** | **Mouse Efficacious Dose (mg/kg)** | **Predicted human Dose by BSA (mg/kg)** | **Xenograft Model (Cell Line)/ Preclinical Dose** | **Preclinical Data Source** | **Ratio** |
| Pomalidomide (Pomalyst) | 4 mg PO/ Immune/ MM | 0.057 | 2.5 | 0.20 | MM (MM1.S human cells)/ 2.5 mg/kg PO | Das et al 2015 | 3.56 |
| Lenalidomide (Revlimid) | 25 mg PO/ Immune/ MM and MCL | 0.36 | 25 | 2.03 | MM (MM1.S human cells)/  25 mg/kg PO | Ocio et al 2015 | 5.63 |
| lurbinectedin (Zepzelca) | 3.2 mg/m^2^ IV/ alkylating and immune/ SCLC | 0.09 | 0.2 | 0.02 | Ovarian  (MNB-PTX1 human cells)/  0.2 mg/kg IV | Romano et al 2013 | 0.19 |
| Tamoxifen (Nolvadex) | 20-40 mg PO/ Hormonal/ Breast | 0.43 | 1.00 | 0.08 | Breast (T47D human cells)/ 25ug/0.025 kg mouse IP | Yeh et al 2014 | 0.19 |
| Toremifene (Fareston) | 60 mg PO/ Hormonal/ Breast | 0.86 | 8.00 | 0.65 | Breast (ZR-75-1 human cells)/  8 mg/kg PO | Gutman et al 2002 | 0.75 |
| Enzalutamide (Xtandi) | 160 mg PO/ Hormonal/ Prostate | 2.29 | 10 | 0.81 | Prostate (LNCaP human cells)/  10 mg/kg PO | Guerrero et al 2013 | 0.35 |
| Flutamide (Eulexin) | 250 mg TID PO/ Hormonal/ Prostate | 10.70 | 10 | 0.81 | Prostate (PDX)/  10 mg/kg PO | Cha et al 2011 | 0.08 |
| Bicalutamide (Casodex) | 50 mg PO/ Hormone antag/ Prostate | 0.71 | 10 | 0.81 | Prostate (VCAP human cells)/ 10 mg/kg PO | Myung et al 2013 | 1.14 |
| Estramustine (Emcyt) | 14 mg/kg PO/ Antiandrogen/  Prostate | 14.00 | 12 | 0.97 | Prostate (PAC120 human cells)/ 12 mg/kg IP | Dahmani et al 2009 | 0.07 |
| BID: twice daily; IP: intraperitoneal(ly); IV: intravenous(ly); MCL: mantle cell lymphoma; MM: multiple myeloma; PO: oral(ly); SCLC: small cell lung cancer; TID: three times daily.  Cha J, Roomi MW, Ivanov V, Kalinovsky T, Niedzwiecki A, Rath M. (2011). Ascorbate depletion increases growth and metastasis of melanoma cells in vitamin C deficient mice. Exp Oncol 33(4):226-30.  Dahmani A, De Plater L, Guyader C, Fontaine J-J, Berniard A, Assayag F, et al. (2009). Abstract A27: efficacy of estramustine + docetaxel in docetaxel-resistant human prostate cancer xenograft: a preclinical model of docetaxel resistance reversion. Mol Cancer Ther. 8(12 Suppl):A27. doi: 10.1158/1535-7163  Das DS, Ray A, Song Y, Richardson P, Trikha M, Chauhan D, et al. (2015). Synergistic anti-myeloma activity of the proteasome inhibitor marizomib and the IMiD immunomodulatory drug pomalidomide. Br J Haematol. 171(5):798-812.  Guerrero J, Alfaro IE, Gómez F, Protter A, Bernales S. (2013). Enzalutamide, an androgen receptor signaling inhibitor, induces tumor regression in a mouse model of castration-resistant prostate cancer. Prostate. 73(12):1291-305. doi: 10.1002/pros.22674.  Gutman M, Couillard S, Roy J, Labrie F, Candas B, Labrie C. (2002). Comparison of the effects of EM-652 (SCH57068), tamoxifen, toremifene, droloxifene, idoxifene, GW-5638 and raloxifene on the growth of human ZR-75-1 breast tumors in nude mice. Int J Cancer. 99(2):273-8. doi: 10.1002/ijc.10302.  Myung J-K, Banuelos CA, Garcia Fernandez J, Mawji NR, Want J, Tien AH, et al. (2013). An androgen receptor N-terminal domain antagonist for treating prostate cancer. J Clin Invest. 123(7):2948-60. doi: 10.1172/JCI66398.  Ocio EM, Fernández-Lázaro D, San-Segundo L, López-Corral L, Corchete LA, Gutierrez NC, et al. (2015). In vivo murine model of acquired resistance in myeloma reveals differential mechanisms for lenalidomide and pomalidomide in combination with dexamethasone. Leukemia. 29(3):705-14. doi: 10.1038/leu.2014.238.  Romano M, Frapolli R, Zangarini M, Bello E, Porcu L, Galmarini CM, García-Fernández LF, et al. (2013). Comparison of in vitro and in vivo biological effects of trabectedin, lurbinectedin (PM01183) and Zalypsis^®^ (PM00104). Int J Cancer. 133(9):2024-33. doi: 10.1002/ijc.28213.  Yeh W-L, Lin H-Y, Wu H-M, Chen D-R (2014) Combination Treatment of Tamoxifen with Risperidone in Breast Cancer. PLoS ONE 9(6): e98805. doi:10. 1371/journal.pone.0098805. | | | | | | | |
